# Computational design of highly signalling-active membrane receptors through solvent-mediated allosteric networks

**DOI:** 10.1038/s41557-024-01719-2

**Published:** 2025-01-23

**Authors:** K.-Y. M. Chen, J. K. Lai, L. S. P. Rudden, J. Wang, A. M. Russell, K. Conners, M. E. Rutter, B. Condon, F. Tung, L. Kodandapani, B. Chau, X. Zhao, J. Benach, K. Baker, E. J. Hembre, P. Barth

**Affiliations:** 1 Institute of Bioengineering, Swiss Federal Institute of Technology (EPFL), Lausanne, Switzerland; 2 Department of Pharmacology, Baylor College of Medicine, Houston, TX USA; 3 Lilly Biotechnology Center San Diego, San Diego, CA USA; 4 Lilly Research Laboratories, Lilly Corporate Center, Indianapolis, IN USA; 5 Present Address: Department of Cell Biology and Human Anatomy, University of California at Davis, Davis, CA USA; 6 Present Address: Tessella, Houston, TX USA; 7 Present Address: AbbVie, North Chicago, IL USA

**Keywords:** Molecular conformation, Membrane proteins, Protein design

## Abstract

Protein catalysis and allostery require the atomic-level orchestration and motion of residues and ligand, solvent and protein effector molecules. However, the ability to design protein activity through precise protein–solvent cooperative interactions has not yet been demonstrated. Here we report the design of 14 membrane receptors that catalyse G protein nucleotide exchange through diverse engineered allosteric pathways mediated by cooperative networks of intraprotein, protein–ligand and –solvent molecule interactions. Consistent with predictions, the designed protein activities correlated well with the level of plasticity of the networks at flexible transmembrane helical interfaces. Several designs displayed considerably enhanced thermostability and activity compared with related natural receptors. The most stable and active variant crystallized in an unforeseen signalling-active conformation, in excellent agreement with the design models. The allosteric network topologies of the best designs bear limited similarity to those of natural receptors and reveal an allosteric interaction space larger than previously inferred from natural proteins. The approach should prove useful for engineering proteins with novel complex protein binding, catalytic and signalling activities.

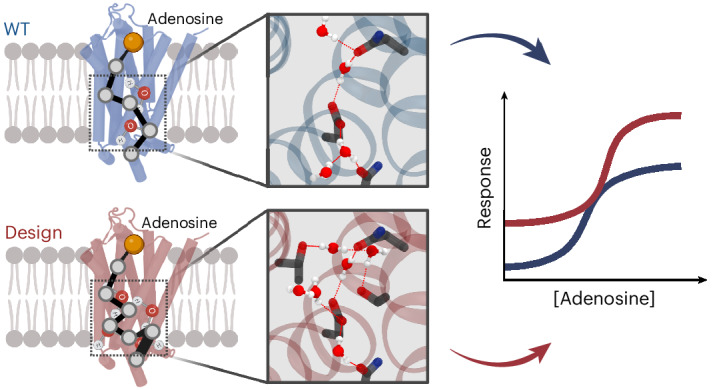

## Abstract (web summary)

Solvent-mediated interaction networks control a wide range of protein functions, but their design has been neglected owing to a lack of accurate computational tools. Now it is shown that allosteric signalling membrane proteins can be engineered through such networks, revealing a broader space of designable protein interactions and functions.

## Main

The remarkable efficiency and selectivity of natural protein catalysts, binders and signalling receptors stem from a delicate balance between stabilizing structural and destabilizing functional motifs. For example, exposed hydrophobic surfaces in soluble proteins encode binding affinity and specificity but can trigger protein aggregation. Enzymes often bear energetically unfavourable networks of buried polar residues critical for substrate binding and catalytic activity. Membrane proteins perform sophisticated signalling and transport functions by rapidly switching between conformations and enabling long-range allosteric communication^1^. Wet transmembrane helical (TMH) interfaces, where solvent molecules bridge destabilizing buried polar residues, facilitate TMH movements by preventing the breaking of hydrogen bond networks^2–4^, granting conformational flexibility. Conversely, buried ion molecules lock receptors in selective conformations through strong electrostatic interactions^5^. Thus, cooperative protein–solvent interaction networks are key universal principles of protein function.

Recent computational methods have incorporated features of natural protein catalysts and binders into designs, achieving high structural specificity and stability in helical bundles through de novo geometrically optimized hydrogen bond networks^6,7^. Key structural features of natural enzymes, including hydrophobic cavities, kinked helices and curved sheets, can now be combined to create novel protein scaffolds^8,9^. These approaches enable an unprecedented level of control over biomolecular shapes and interactions. However, the design of new protein activities that balance stabilizing and destabilizing features remains a major challenge.

G protein-coupled receptors (GPCRs) constitute the largest family of signalling membrane receptors and drug targets. Built on a common seven-transmembrane (7TM) helical scaffold, they sense a wide range of extracellular stimuli (for example, ions, lipids, proteins and small-molecule ligands) to activate distinct intracellular signalling pathways mediated by G proteins and arrestins. Structural studies of receptors in different functional states (for example, ‘inactive’, where receptors are bound to antagonist or inverse agonist ligands, ‘partially active’, where receptors are bound to agonist ligands, and ‘active’, where receptors are bound to agonist ligands and intracellular effector proteins) have highlighted common structural changes upon activation, ranging from individual side-chain conformational shifts (that is, microswitches) to large rigid-body TMH movements. The receptor core, connecting the extracellular ligand and intracellular effector binding sites, bears several highly conserved polar residues connected through buried solvent and ion molecules. This network undergoes considerable changes upon receptor activation^10–12^, suggesting its important participation in the allosteric transitions underlying signal transduction. Notably, a sodium ion has been shown to preferentially bind to the highly conserved Asp2.50 (designated according to the Ballesteros–Weinstein notation) in the inactive state, thereby acting as a negative allosteric modulator^5,13^. These observations make GPCRs a suitable model system for studying and testing through design our understanding for how solvation underpins protein structure and function.

Here, we report a computational approach to the design of proteins with novel stability and signalling functions through engineered cooperative intraprotein, protein–ligand and –solvent molecule interaction networks. Beginning with the adenosine A2A receptor (A2AR) scaffold, we designed membrane receptors with varying signalling activity and thermostability. In close agreement with the design models, a highly signalling-active designed receptor crystallized in an atypical GPCR active-like conformation despite the absence of a bound G protein. Our results reveal that signalling activity and thermostability can be engineered concurrently through a careful rewiring of reprogrammable ligand- and solvent-mediated allosteric interaction networks. Our approach should prove useful for engineering proteins with novel catalytic, signal transduction and allosteric functions.

## Results

### Design principles of solvent-mediated allosteric networks

**Fig. 1:**
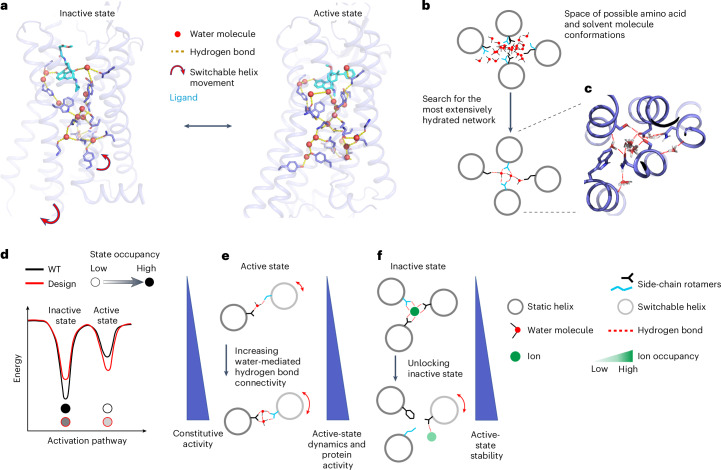
Rational design of signalling activity through de novo solvent-mediated interaction networks. **a**, Dynamic remodelling of the solvent-mediated allosteric network during the activation of the μ-opioid receptor (mOR). The positions of water molecules and hydrogen bonds were extracted from the inactive (Protein Data Bank (PDB): 4DKL) and active (PDB: 5C1M) mOR structures. **b**, Schematic representation of a water-mediated hydrogen bond network bridging four helices designed using the SPaDES software. De novo amino acid sequences, conformations and water positions were searched concurrently for optimal water-mediated hydrogen bond connectivity at the helical interface. **c**, Snapshot of a low-energy water-mediated hydrogen bond network ensemble in the core of a GPCR structure. **d**–**f**, Computational design strategies for enhancing protein activity and conformational stability following three criteria: shifts in conformational energies (**d**), increased water-mediated dynamic contacts in the active state (**e**) or weakened ion-mediated locks in the inactive state (**f**).

Protein catalysis and allostery often rely on the concerted motions of flexible regions that switch between distinct conformations around a stable and rigid scaffold (Fig. 1a). We define the interactions between rigid and flexible structures as ‘static–switchable’. Water molecules can facilitate such movements by bridging polar residues to create extensive dynamic interaction networks. Conversely, ion molecules tend to stabilize specific conformations through strong electrostatic interactions at the expense of protein flexibility. Following these observations, we reasoned that signalling activity could be programmed into protein scaffolds by designing solvent-mediated interaction networks that connect the static–switchable TMH–TMH interfaces critical for activation.

To facilitate the design of such properties, we developed a computational method that can build protein interiors with customized solvent-mediated interaction networks. The method first models receptor conformations in specific (for example, inactive and active) functional states by homology to native protein structures with related functions using the software IPHoLD^14^. In these models, TMH regions undergoing substantial conformational changes upon receptor activation are referred to as ‘switchable’, while those remaining largely unperturbed are described as ‘static’. The interfaces between the static and switchable TMH regions are then designed using the SPaDES software, developed to model and design protein structures and interactions with explicit solvent^15^. We used SPaDES to search for combinations of amino acids and associated solvent molecules that form highly coordinated networks of solvent-mediated interactions between the TMHs in both the inactive and active states (Fig. 1b,c). Following our above-mentioned hypothesis, designs were selected on the basis of three criteria. (1) The overall conformational stability of the active state compared with that of the inactive state, calculated as the energy difference of the protein in the two states (Fig. 1d and Methods). This criterion can be translated into the relative occupancy of the protein in each state and therefore be predictive of changes in constitutive activity. (2) The level of water-mediated hydrogen bond connectivity between static and switchable TMHs. Increased interface plasticity via high levels of hydration should facilitate motion and activation of the receptor upon agonist ligand binding. Hence, we expect this feature to be predictive of receptor signal transduction and G protein activation propensity. The topologies and energies of water-mediated hydrogen bond interaction networks were calculated using graph analysis and SPaDES, respectively (Fig. 1e and Methods). (3) The strength of protein–ion interactions locking and stabilizing the protein into a specific conformation. Ion–protein interactions were calculated through cycles of coarse and fine-grained ion placement grid sampling within the protein interior, followed by hydration and a complete repack and minimization of the protein conformation using SPaDES (Fig. 1f and Methods). In our model, preferential ion binding to specific states should modulate conformational stability, state occupancy and hence protein activity. Overall, sequence space was searched primarily through the modulation of criterion 1 (for example, through a decrease or increase in the conformational energy difference), while criteria 2 and 3 were used to rank and select designs before experimental validation.

### Computational design of GPCRs with reprogrammed activity

**Fig. 2:**
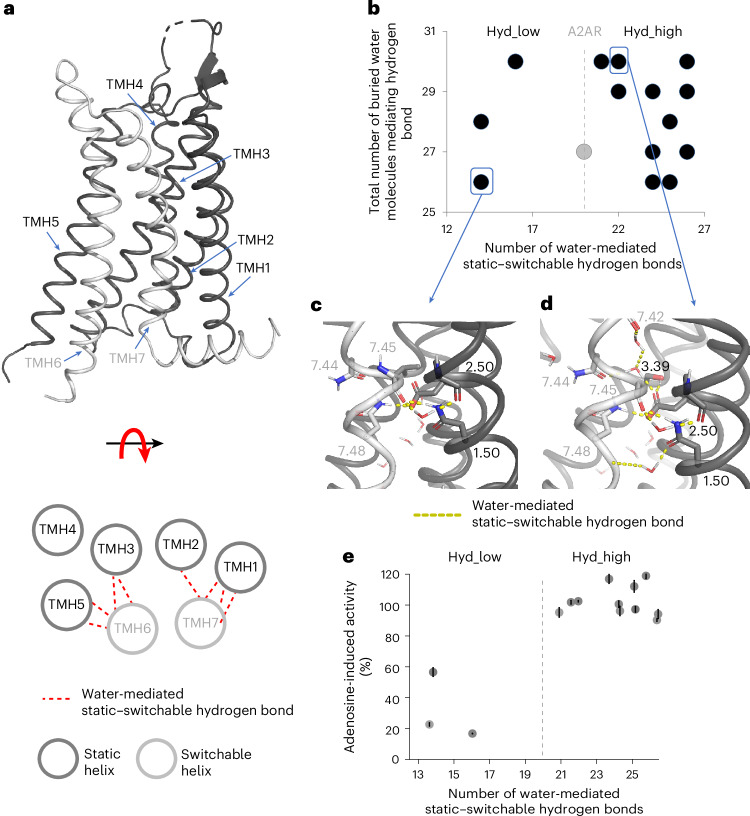
Computational-designed GPCRs with a wide range of water-mediated interaction networks at switchable TMH interfaces. **a**, Backbone representation of a GPCR scaffold highlighting static (dark grey) and switchable (light grey) TM helices. **b**, Relationship between the total number of buried water molecules mediating polar contacts and those at static–switchable TMH interfaces. Data for the native A2AR (light grey) are included for comparison. Designs with a higher and lower number of water-mediated static–switchable hydrogen bonds than A2AR are classified as Hyd_high and Hyd_low, respectively. **c**,**d**, Hydrogen bond network in the TMH core regions of the Hyd_low2 (**c**) and Hyd_high4 (**d**) structures. The positions of the residues highlighted as stick models are indicated by Ballesteros–Weinstein notation. **e**, Adenosine-induced activity of the designed receptor variants (as a percentage of adenosine-induced WT A2AR activity) as a function of the number of solvent-mediated interactions at static–switchable TMH interfaces. A baseline for the native A2AR (light grey) is included for comparison. The data are presented as the mean ± standard error of the mean (s.e.m.) for *n* = 3 independent experiments. Source data

As a proof of concept, we applied the approach to programme signalling activity into an adenosine-sensing GPCR. The 7TM region transmits extracellular ligand-induced signals through the specific concerted motions of two main helices (TMH6 and TMH7) that undergo substantial structural changes to trigger the opening of a G protein binding site on the intracellular side of the receptor (Fig. 2a)^16–19^. We define TMHs 6 and 7 as ‘switchable’ and TMHs 1–5, whose conformations remain very similar in distinct functional states, as ‘static’. Inactive- and active-state adenosine-sensing GPCR models were constructed from the antagonist-bound A2AR X-ray structures and the active-state X-ray structure of beta 2 adrenergic receptor (B2AR) bound to the G protein Gs, respectively using IPHoLD^14^. We then applied SPaDES to design new sequences and solvent-mediated interactions at the interface between the static TMHs 1, 2, 3 and 5 and the switchable TMHs 6 and 7. In this approach, the ligand and G protein binding sites were not subjected to design. A total of 22 buried positions and associated solvent molecules were designed in the receptor. Due to the computational overhead associated with SPaDES, we restricted the sequence search space to hydrophobic, uncharged polar residues and small charged amino acids compatible with folding and/or high packing constraints in the TM core (Methods). Receptors were designed through modulation (that is, a decrease or increase) of the conformational stability between their inactive- and active-state structures (criterion 1) and selected variants were analysed for their water-mediated interaction network topologies at static–switchable interfaces (criterion 2) and conformation-specific protein–ion interactions (criterion 3; Supplementary Tables 1–3 and Methods). Nine single-point mutant variants and five variants with combinatorial mutations at multiple sites were ultimately selected for experimental characterization to assess the impact of sequence variations at diverse sites, including highly conserved positions. These provided a stringent test of the hypothesis underlying our approach and our design criteria.

The designed receptors were expressed in mammalian cells, purified and tested in vitro for constitutive, adenosine ligand-induced activation of the G protein Gs, ligand binding and active-state stability (Methods and Supplementary Fig. 1). From these measurements, we examined (1) the equilibrium between the ligand-free inactive and active states, (2) the ligand-induced activation and hence the allosteric coupling between the ligand and G protein binding sites, (3) the receptor–ligand binding affinity and (4) the stability of the active-state conformation. These properties were sufficient to assess the impact of the designs on the allosteric signalling properties of the receptor within a classic allosteric two-state model^17^.

We first measured Gs activation upon ligand titration and observed two classes of response. While three designs displayed considerably impaired activity, the other eleven showed similar or higher activity than A2AR (Fig. 2, Supplementary Fig. 2 and Supplementary Table 4). Analysis of the solvent-mediated interaction network elucidated the origin of this functional diversity. While all of the designed cores possessed a high number of water-mediated hydrogen bonds (up to 28), we found significant correlations between activity and the number of solvated static–switchable interactions (between 14 and 26; Fig. 2b). For comparison, our simulations showed the native A2AR to have a total of 25 water-mediated hydrogen bonds with 20 static–switchable water-mediated interactions. We denote designs with a lower and higher density of solvated static–switchable interactions than A2AR as Hyd_low and Hyd_high, respectively (Fig. 2b–d and Supplementary Table 2). Remarkably, all of the Hyd_low designs triggered lower adenosine-induced activity than A2AR (between 10% and 60% of A2AR activity), while all of the Hyd_high designs showed similar or higher (up to 123%) activities (Fig. 2e). These results validate the hypothesis underlying our second design criterion and suggest that water-mediated interactions at static–switchable TMH interfaces are strong determinants of receptor allosteric signalling responses.

**Fig. 3:**
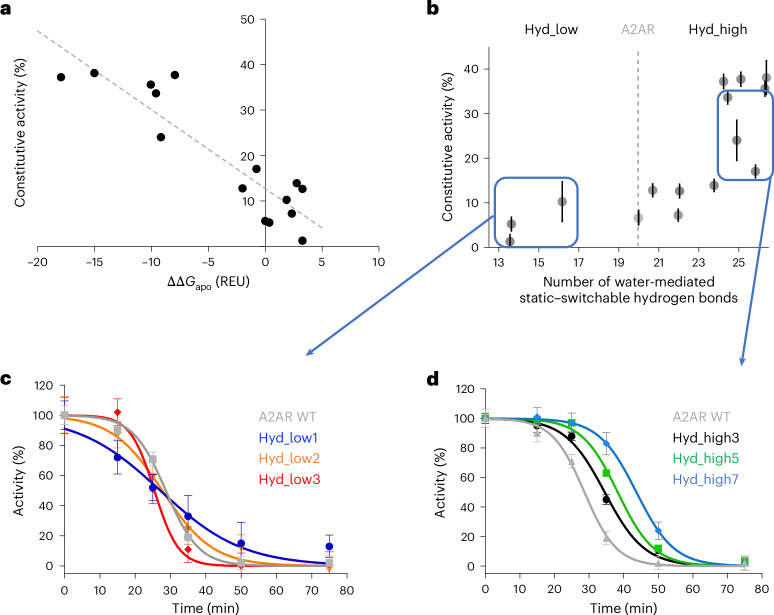
Designed receptor stability and function strongly depend on the density of solvent-mediated interactions at switchable TMH interfaces. **a**, Constitutive activity (as a percentage of maximal agonist-induced WT A2AR activity) as a function of the calculated conformational energy difference between the active and inactive states relative to A2AR. The reported energy difference was calculated from the average of the ten lowest-energy inactive- and active-state structures (Supplementary Table 3). The best linear fit to the data is shown as a dotted line (correlation coefficient *r* = 0.91). **b**, Measured constitutive activity of the designed receptor variants (as a percentage of adenosine-induced A2AR activity) as a function of the number of solvent-mediated interactions at static–switchable TMH interfaces. **c**,**d**, Apparent stability of Hyd_low (**c**) and Hyd_high (**d**) receptors compared with A2AR, measured as the functional half-life of adenosine-bound receptor variants incubated at 37 °C. The values for all of the designs are given in Supplementary Table 4. In **b**–**d**, the data are presented as the mean ± s.e.m. for *n* = 3 independent experiments. Source data

We then measured receptor constitutive activities in the absence of ligand. The three Hyd_low designs displayed similar or lower constitutive activities than A2AR, while the spontaneous activities of the eleven Hyd_high designs were notably higher, ranging from 7% to 38% of the maximal adenosine-induced A2AR activity. The constitutive activities correlated strongly with the conformational stabilities calculated in the absence of ligand (ΔΔ*G*_apo_), in agreement with our first design criterion and the allosteric two-state model (Fig. 3a, Supplementary Table 4 and Supplementary Fig. 3). Consistent with a higher active-state occupancy, the elevated constitutive activities measured for the Hyd_high designs were corroborated by an enhanced binding affinity for the ligand agonist adenosine (Supplementary Table 5). We also directly assessed receptor conformational stability by measuring receptor activity as a function of incubation time at 37 °C. While the three variants with low constitutive activity rapidly lost their function, the active-state stability of the other eleven designs was higher than that of A2AR, with half-lives reaching as long as 44 min compared with 29 min for the native receptor (Fig. 3a–c and Supplementary Table 4). Lastly, the receptors displayed a wide range of ion binding properties that are highly specific to the structural context of each variant and are described below for the combinatorial designs. Overall, the results largely validate our design approach and suggest that SPaDES captures important determinants of conformational stability, including those mediated by solvent interactions.

### Mechanistic insights into solvent-mediated allostery

Beyond the above-described global features that enabled classification of the designs and overall validation of the design strategy, we observed striking relationships between solvent-mediated allosteric networks and receptor activities that provide mechanistic insights into the role of solvent in GPCR signal transduction.

**Fig. 4:**
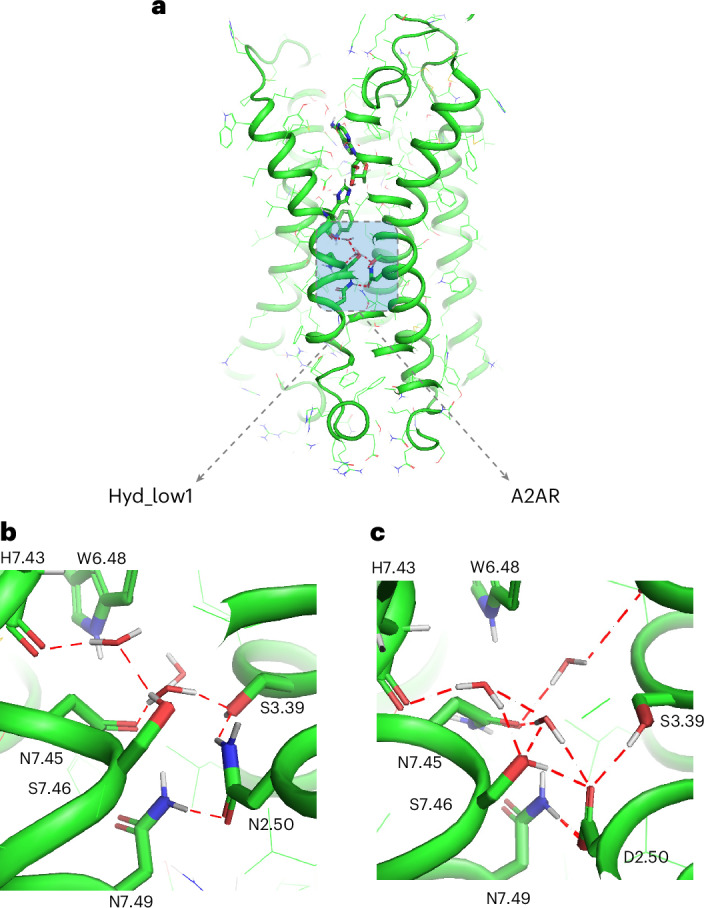
Polar interaction networks connecting static and switchable TMHs in the Hyd_low1 design and A2AR. **a**, Cartoon representation of the Hyd_low1 receptor active-state model, where the adenosine ligand and side chains in the vicinity of the designed mutations are highlighted as stick models. **b**,**c**, Enlarged views of the designed region in Hyd_low1 (**b**) and A2AR (**c**). Polar contacts are represented by red dotted lines. Residues are numbered using the Ballesteros–Weinstein notation.

We first assessed the impact of designed amino acid substitutions at the highly conserved Asp2.50 located on TMH2. In our calculations, an Asn at that site (the Hyd_low1 design) destabilized the active state by more than 3 Rosetta energy units (REU) and also led to a 30% reduction in water-mediated static–switchable hydrogen bonds (Supplementary Table 2). Consistent with the predictions, we observed a lower constitutive activity, a slight drop in the active-state half-life and a large decrease in adenosine-induced activity compared with A2AR (Supplementary Table 4). The loss of water-mediated interactions primarily affected critical contacts stabilizing TMH7 distortions (involving His7.43, Asn7.45, Ser7.46 and Asn7.49) above the NPxxY motif that also facilitate TMH7 motion during activation, providing a rationale for the impact on receptor activation (Fig. 4).

**Extended Data Fig. 1:**
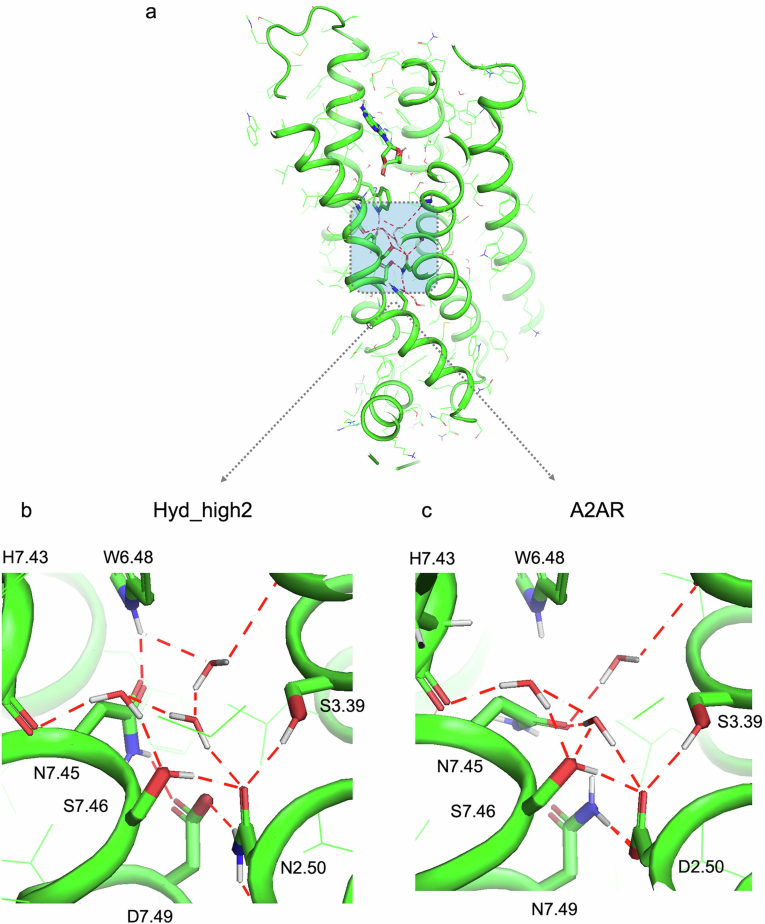
Polar interaction networks connecting static and switchable TMHs in the Hyd_high2 design and A2AR. **a**, Cartoon representation of the Hyd_high2 receptor active state model where the adenosine ligand and side-chains in the vicinity of the designed mutations are highlighted in sticks. **b,c**, zoomed view on the designed region in Hyd_high2 (b) and A2AR (c). Polar contacts are represented with red dotted lines. Residues are numbered using the Ballesteros Weinstein notation.

We next reasoned that the Hyd_high2 design, bearing the Asn7.49Asp mutation of the conserved neighbouring residue, could rescue the Hyd_low1 design. Swapping the native Asn and Asp tests the impact of the position and structural context of polar residues on receptor stability and function. The introduction of Asp into TMH7 considerably increased the hydration, enabling the formation of 20% more water-mediated static–switchable hydrogen bonds than in A2AR (Supplementary Table 2). Consistent with this effect, the maximal agonist-induced activity was 17% higher than A2AR, indicating that potent signalling functions were restored in Hyd_high2. Increased constitutive activity and active-state half-life were also observed compared with Hyd_low1 (Supplementary Table 4). While fully functional, the water-mediated interaction network was strongly perturbed by the designed mutations. The conserved Asn7.45 underwent a large conformational change compared with A2AR, abolishing its water-mediated contacts with TMH2 (through position 2.50) and TMH6. Instead, Asn7.45 established a direct hydrogen bond with the indole group of Trp6.48. In addition, two water molecules near the sites 2.50 and 7.49 changed conformation to accommodate the mutations and maintain strong water-mediated interactions between TMH2 and TMH1 (through Asn1.50, Extended Data Fig. 1). These conformational switches also rewired the interaction network between TMH2 and TMH7 through novel water-mediated contacts between Asn2.50 and His7.43.

Overall, the Hyd_low1 and Hyd_high2 designs reveal that Asp2.50 is a major orchestrator of hydration between TMHs 1, 2, 3 and 7 in the buried core of the receptor active state. Importantly, our interchanged Asp/Asn design suggests that receptor activation is tolerant to the position of charges/polar residues in the buried TM core provided that strong hydration at the static–switchable TMH interface where substantial motion occurs during receptor activation is maintained.

**Extended Data Fig. 2:**
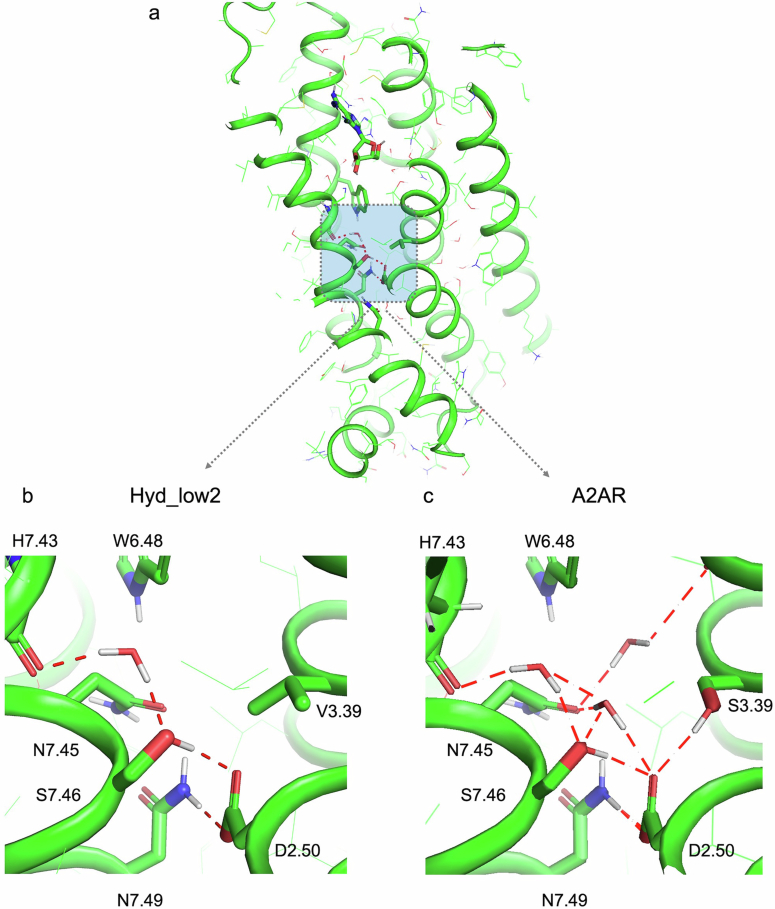
Polar interaction networks connecting static and switchable TMHs in the Hyd_low2 design and A2AR. **a**, Cartoon representation of the Hyd_low2 receptor active state model where the adenosine ligand and side-chains in the vicinity of the designed mutations are highlighted in sticks. **b,c**, zoomed view on the designed region in Hyd_low2 (b) and A2AR (c). Polar contacts are represented with red dotted lines. Residues are numbered using the Ballesteros Weinstein notation.

We then analysed the designs selected at the 3.39 site on TMH3. In A2AR, Ser3.39 interacts directly with Asp2.50 and mediates water-mediated interactions between the static TMH3 and the switchable TMHs 6 and 7. Our calculations selected a valine substitution (Hyd_low2) that abrogated all water-mediated interactions involving Ser3.39 in A2AR. Consequently, the number of water-mediated hydrogen bonds at static–switchable TMH interfaces dropped by 30%, contributing to the substantial loss in our measured ligand-induced activity (57% of A2AR; Supplementary Tables 2 and 4). The critical solvent-mediated contacts between TMHs 2 and 3 and TMHs 6 and 7 that control the motions of TMHs 6 and 7 were also disrupted (Extended Data Fig. 2). The design also abrogated a key direct polar contact between TMHs 2 and 3 that locks the two helices in the active-state conformation. Altogether, these features prevent optimal receptor stabilization in the active state and activation.

**Extended Data Fig. 3:**
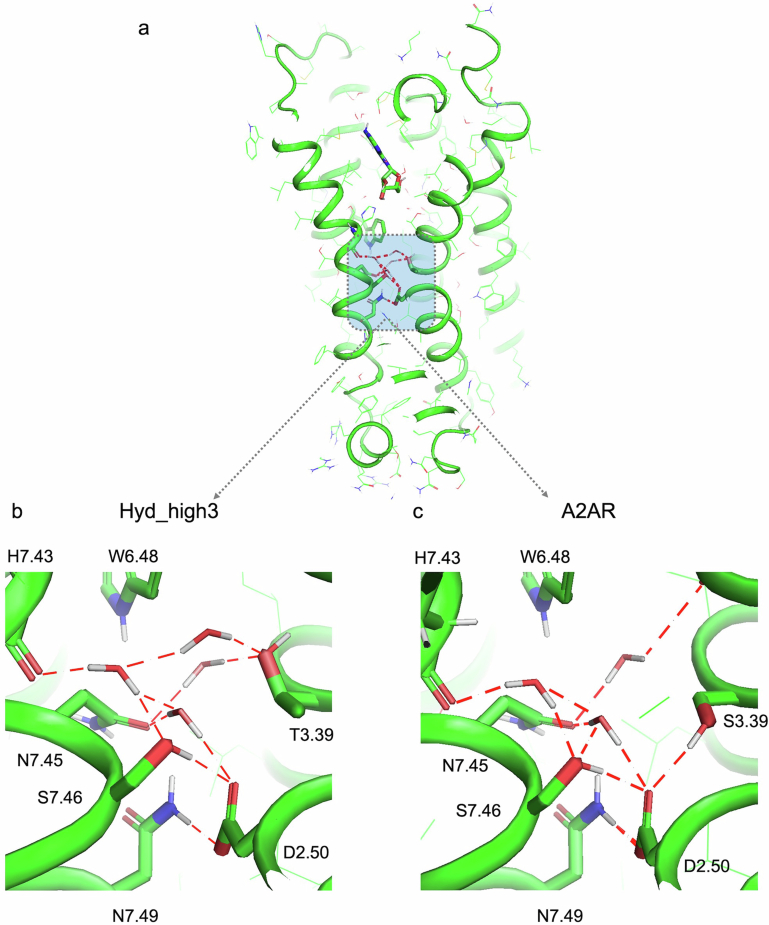
Polar interaction networks connecting static and switchable TMHs in the Hyd_high3 design and A2AR. **a**, Cartoon representation of the Hyd_high3 receptor active state model where the adenosine ligand and side-chains in the vicinity of the designed mutations are highlighted in sticks. **b,c**, zoomed view on the designed region in Hyd_high3 (b) and A2AR (c). Polar contacts are represented with red dotted lines. Residues are numbered using the Ballesteros Weinstein notation.

To further understand the impact of steric and polarity variations at site 3.39, we next examined Thr3.39 (Hyd_high3). While maintaining the same polarity as native Ser3.39, the design substantially increased the number of water-mediated static–switchable TMH interactions (26 versus 20, Supplementary Table 2) owing to a different position of the side-chain hydroxy group. This conformational microswitch considerably perturbed the solvent-mediated network in the cavity. Thr3.39 no longer directly interacts with Asp2.50, instead mediating new strong water-mediated contacts with His7.43 and Asn7.45. Overall, we observed a shift of polar contacts with a higher number of interactions between TMH3 and TMH7 (Extended Data Fig. 3). Despite the substantial rewiring of the allosteric interaction network, adenosine-induced activity was enhanced (close to 120% of A2AR), consistent with a 30% increase in hydration at static–switchable interfaces (Supplementary Table 4). In addition, our calculations pointed towards an overall enhanced conformational stability of the active state, which we verified experimentally (active-state half-life of 34.6 min compared with 28.7 min for A2AR, Supplementary Table 4). Overall, our results indicate that both Asp2.50 and position 3.39 critically shape the allosteric interaction network through static and switchable interactions between TMH3 and the neighbouring TMHs 2 and 7.

**Extended Data Fig. 4:**
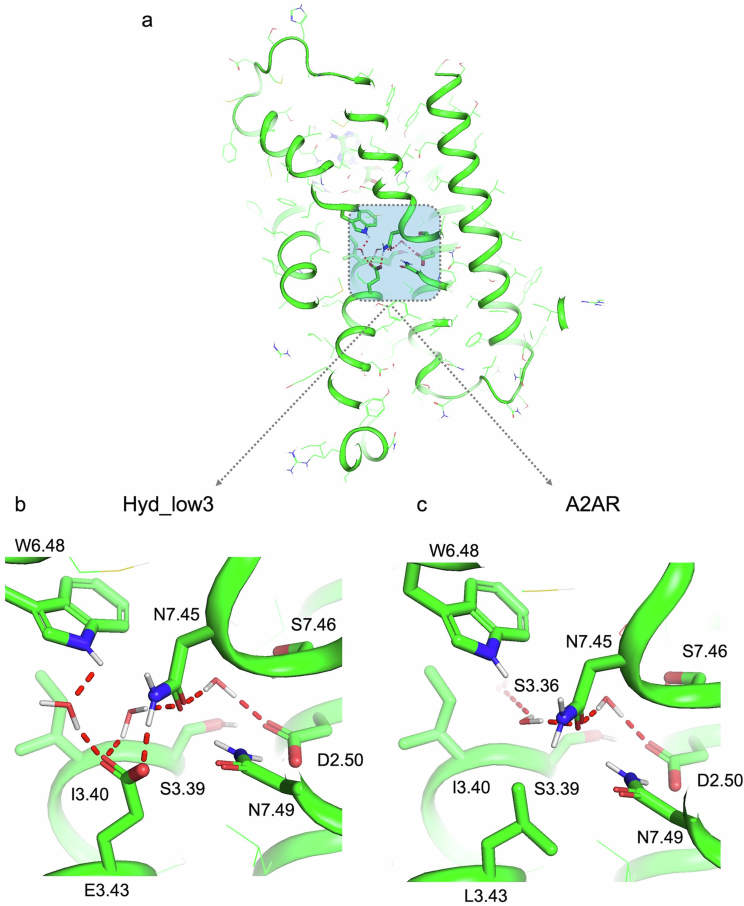
Polar interaction networks connecting static and switchable TMHs in the Hyd_low3 design and A2AR. **a**, Cartoon representation of the Hyd_low3 receptor active state model where the adenosine ligand and side-chains in the vicinity of the designed mutations are highlighted in sticks. **b,c**, zoomed view on the designed region in Hyd_low3 (b) and A2AR (c). Polar contacts are represented with red dotted lines. Residues are numbered using the Ballesteros Weinstein notation.

**Extended Data Fig. 5:**
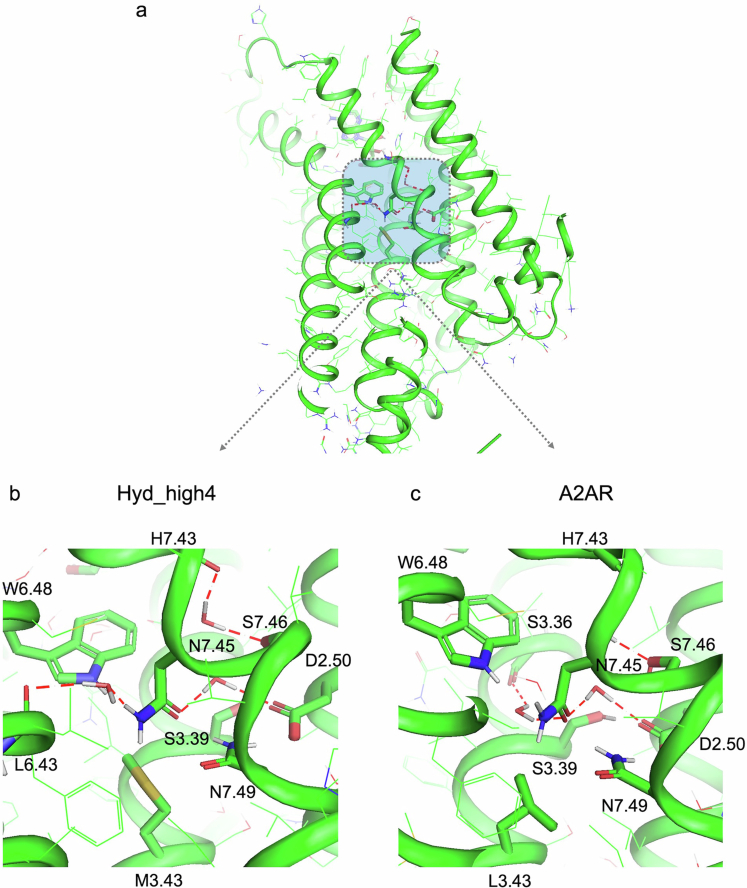
Polar interaction networks connecting static and switchable TMHs in the Hyd_high4 design and A2AR. **a**, Cartoon representation of the Hyd_high4 receptor active state model where the adenosine ligand and side-chains in the vicinity of the designed mutations are highlighted in sticks. **b,c**, zoomed view on the designed region in Hyd_high4 (b) and A2AR (c). Polar contacts are represented with red dotted lines. Residues are numbered using the Ballesteros Weinstein notation.

**Extended Data Fig. 6:**
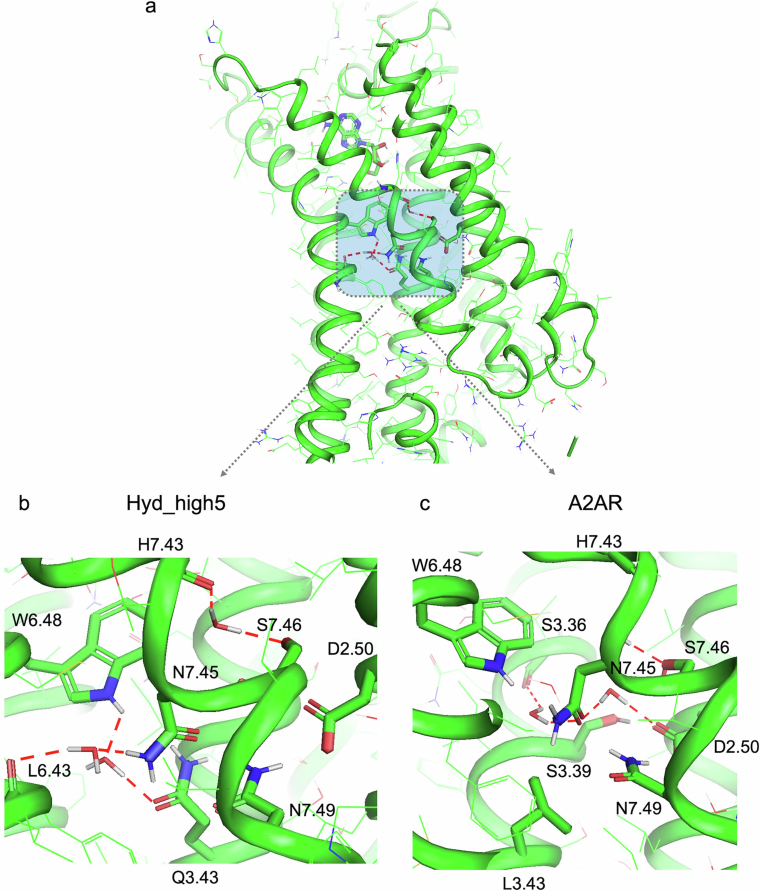
Polar interaction networks connecting static and switchable TMHs in the Hyd_high5 design and A2AR. **a**, Cartoon representation of the Hyd_high5 receptor active state model where the adenosine ligand and side-chains in the vicinity of the designed mutations are highlighted in sticks. **b,c**, zoomed view on the designed region in Hyd_high5 (b) and A2AR (c). Polar contacts are represented with red dotted lines. Residues are numbered using the Ballesteros Weinstein notation.

**Extended Data Fig. 7:**
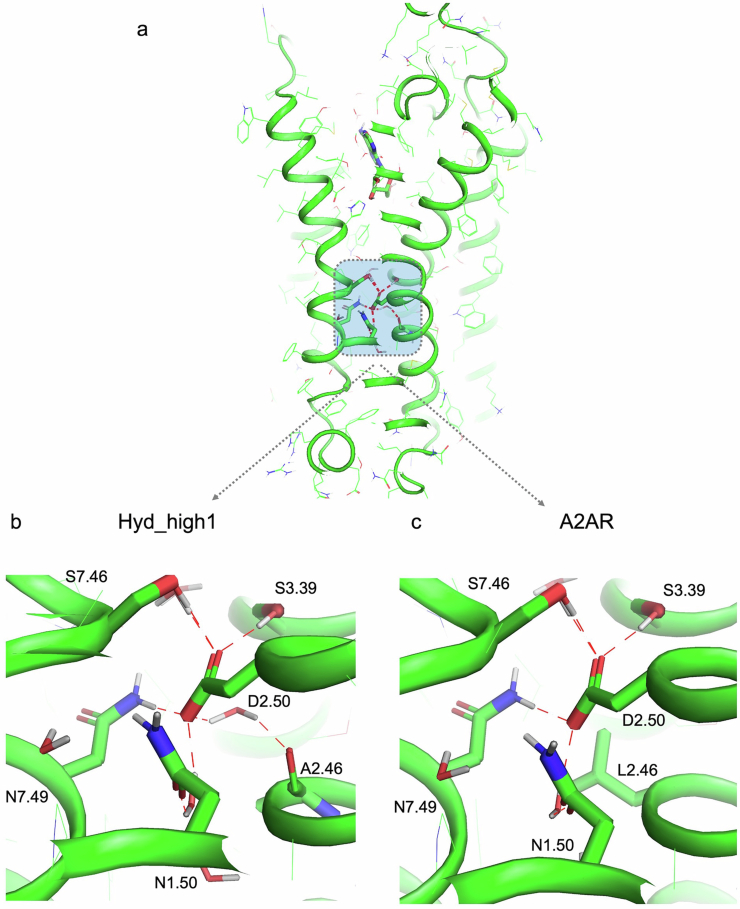
Polar interaction networks connecting static and switchable TMHs in the Hyd_high1 design and A2AR. **a**, Cartoon representation of the Hyd_high1 receptor active state model where the adenosine ligand and side-chains in the vicinity of the designed mutations are highlighted in sticks. **b,c**, zoomed view on the designed region in Hyd_high1 (b) and A2AR (c). Polar contacts are represented with red dotted lines. Residues are numbered using the Ballesteros Weinstein notation.

We next investigated the role of hydrophobic residues lining the hydrated cavity in the solvent-mediated polar network and signal transduction by targeting positions 2.46 and 3.43 located one helical turn below Asp2.50 and Ser3.39, respectively (Supplementary Discussion and Extended Data Figs. 4–7). Most mutations substantially disturbed the water-mediated allosteric network topology through the creation of microcavities or removal of the hydrophobic gateway that separates the solvated cavity from the active-state intracellular side. However, except for the Leu3.43Glu variant, we did not observe any notable loss of functions, in line with our calculations.

**Extended Data Fig. 8:**
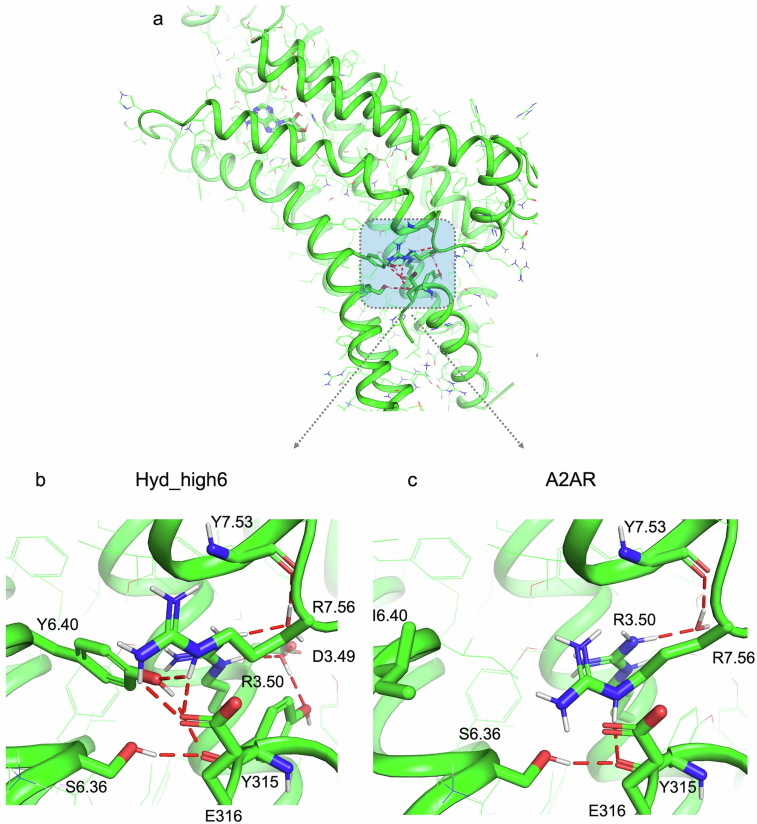
Polar interaction networks connecting static and switchable TMHs in the Hyd_high6 design and A2AR. **a**, Cartoon representation of the Hyd_high6 receptor active state model where the adenosine ligand and side-chains in the vicinity of the designed mutations are highlighted in sticks. **b,c**, zoomed view on the designed region in Hyd_high6 (b) and A2AR (c). Polar contacts are represented with red dotted lines. Residues are numbered using the Ballesteros Weinstein notation. Y315 and E316 belong to the G-protein Gαs.

Lastly, we investigated the possibility of reprogramming the intracellular solvent-mediated interaction network at the binding interface between the receptor and the G protein Gαs. The design Hyd_high6 substitutes Tyr for the native Ile6.40 located at the intracellular tip of TMH6. The side-chain of Tyr6.40 reaches out to the G protein and establishes stronger polar contacts with Glu316 of Gαs. In turn, Arg7.56 conformational switching generates space for a new water-mediated contact between the highly conserved Arg3.50, Asp3.49 from the DRY motif and Tyr315, stabilizing the active-state conformation of these microswitches (Extended Data Figure 8). Altogether, stronger interactions with Gαs and between activating microswitches should promote complex formation and stabilize the active state even without agonist. These predictions were confirmed by a large increase in measured basal activity (~20%; Supplementary Table 4).

We next considered how combinatorial designs involving multiple TMH core sites might impact conformational stability and allosteric signal transduction through a more extensive network. For our calculations, we selected five designs, Hyd_high7 to Hyd_high11, owing to their high hydration levels. These variants incorporated several combinations of the single-mutant designs, activating features that together were predicted to greatly enhance the conformational stability of the receptor active form. In agreement with the calculations, we observed large increases in constitutive activity (up to 38% of the maximal adenosine-induced A2AR activity) and active-state stability (up to 15 °C in apparent melting temperature) relative to A2AR, while the adenosine-induced activities remained similar to the other Hyd_high designs (Supplementary Table 4). The considerable shift towards the active state resulted in a more than twofold increase in receptor binding affinity for adenosine (Supplementary Table 5).

Structural analysis revealed a number a design-specific effects on the solvent-mediated network topologies and Na^+^ binding propensities. From our Na^+^ binding simulations, we extracted ion occupancies in distinct states of the receptors and validated them using thermal shift assays in the presence of either Na^+^ or K^+^, where K^+^ is not well accommodated in the Na^+^ binding site of GPCRs. Overall, we observed qualitative agreement between our ion binding predictions and the experiments, suggesting that our approach captures the important determinants of conformationally selective receptor–ion interactions (Supplementary Discussion, Methods, Supplementary Table 6 and Supplementary Fig. 4).

Altogether, the measured high constitutive and ligand-induced activities associated with enhanced active-state stability and agonist ligand binding affinity provide direct evidence that our design strategy has maintained or even improved the allosteric coupling underlying signal transduction despite the substantial rewiring of the solvent-mediated network in the Hyd_high designs.

Overall, five striking new features associated with GPCR signal transduction and solvent-mediated allostery emerged from the analysis of the designs.

1. The density of the water-mediated contacts between static TMHs 2 and 3 and switchable TMHs 6 and 7 is a primary determinant for ligand-induced signal transduction.
2. Even minor sequence variations can profoundly impact the solvent-mediated TMH–TMH network topology, further indicating that receptor sequence polymorphism can readily alter receptor signalling.
3. Several conserved polar residues, such as Asp2.50 and Ser3.39, are major hubs in the solvent-mediated interaction networks and orchestrate the 7TMH scaffold activating motions.
4. Not all network perturbations are functionally equal. Receptor activation seems largely insensitive to rewiring at Trp6.48 and the hydrophobic gateway. Increasing contacts, swapping polar residues and network topology rewiring are tolerated if polar connections facilitating key conformational changes in TMH7 are maintained. Wet contacts between static TMHs 2 and 3 and switchable TMH7 are critical for facilitating allosteric motions and receptor activation.
5. While primarily controlling allosteric transitions and signal transduction, water- and ion-mediated interactions can also modulate conformational stability and basal signalling.

### Hyd_high7 structure reveals a new receptor active form

**Fig. 5:**
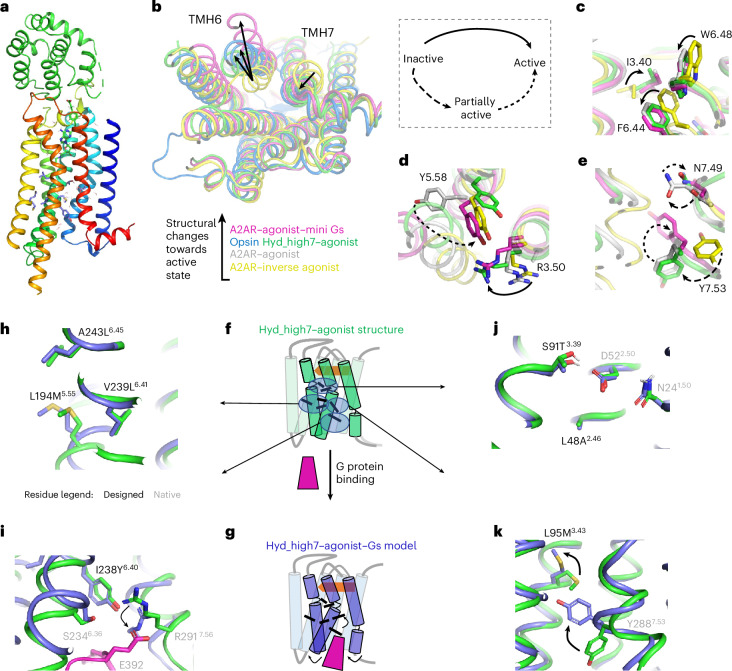
The designed Hyd_high7 structure adopts an unforeseen activated conformation. **a**, Backbone representation of the designed Hyd_high7 structure bound to the ligand agonist CGS21680 and N-terminally fused to a T4Lcp variant. **b**, Cytoplasmic view of the following superimposed structures: antagonist-bound A2AR (PDB: 3EML, yellow), agonist-bound, computationally designed Hyd_high7 (green), Gt peptide-bound opsin active state (PDB: 3DQB, blue), and agonist- and mini-Gs-bound A2AR thermostabilized by alanine scanning (PDB: 5G53, magenta). The structures are ranked according to the magnitude of the conformational changes towards the fully active state (back arrow). **c**–**e**, Conformations of key conserved microswitches involved in receptor activation (in addition to the reference structures described in panel **b**, the following structure is also represented: agonist-bound A2AR thermostabilized by alanine scanning (PDB: 2YDO, grey)): the TMH3–TMH5–TMH6 interface around the W6.48 toggle switch in the receptor TM core (**c**), the TM3–TMH5–TMH6 interface around the intracellular DR^3.50^Y motif (**d**) and the TMH6–TMH7 interface around the intracellular N^7.49^PXXY^7.53^ motif (**e**). Conformational changes between inactive, partially active and fully active states are indicated by distinct black arrows. **f**–**k**, Comparison of the conformation of the designed (black) and neighbouring native (grey) residue microswitches in the agonist-bound designed Hyd_high7 structure (**f**) and the agonist- and Gs-bound Hyd_high7 design model (**g**) as well as the TMH5–TMH6 core interface (**h**), the TMH6–TMH7 intracellular interface with bound Gs (magenta) (**i**), the TMH1–TMH2–TMH3 core interface at the Na^+^ ion-conserved binding site (**j**) and the TMH3–TMH6–TMH7 intracellular interface (**k**).

**Extended Data Fig. 9:**
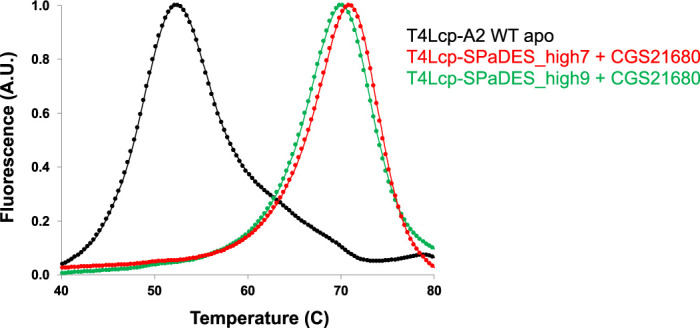
Fluorescent Thermal Stability Assay of T4Lcp- Hyd_high constructs. Measurements were performed for N-terminus circular permutant T4-Lyzozyme - designed Hyd_high 9 (green) and 7 (red) crystallization constructs bound to the ligand agonist CGS21680. Source data

We next sought to validate the structures of our designed receptors. We selected our most stable variants and screened for potent agonists and fusion proteins at the N terminus of the receptor to achieve maximal expression and apparent agonist-bound stability (Methods)^20^. When fused to a circularly permutant T4 lysozyme variant (T4Lcp) and bound to the CGS21680 agonist, Hyd_high7 displayed a large increase in thermodynamic stability compared with the native A2AR (~20°C), reaching an apparent melting temperature of 72 °C (Extended Data Fig. 9 and Supplementary Fig. 5). We successfully crystallized and resolved the CGS21680-bound T4Lcp–Hyd_high7 construct (Fig. 5a, Supplementary Table 7 and Supplementary Fig. 6). While the moderate 3.9 Å resolution prevented the unambiguous assignment of electron density to solvent molecules, the ligand agonist and most side-chain conformations could be readily characterized (Supplementary Figs. 7 and 8). In broad agreement with the designed model, the agonist-bound Hyd_high7 adopted an unforeseen active-like conformation despite the absence of G protein. While the orientation of TMH6 was unique among GPCR active forms, TMH7 adopted a canonical active-state conformation (Fig. 5b)^21^. Similar to the ternary active-state structure of wild-type (WT) A2AR, the presence of the full-length G protein often induces additional distortions in the intracellular tip of TMH6 (Fig. 5b)^22^. GPCR activation involves the side-chain movements of several highly conserved residue microswitches, constituting key molecular signatures of receptor signalling^23,24^. Because a few of these native amino acids are critical for G protein binding, they were introduced into our designed receptors. While these residues usually adopt only a partially active conformation in native agonist-bound GPCRs^25^, they displayed a unique combination of partially active, fully active and unforeseen conformations in the Hyd_high7 structure (Fig. 5c–e). To assess whether the novel conformational state occupied by Hyd_high7 was not biased by extensive crystallization contacts, we analysed the packing contacts in the unit cell (Supplementary Fig. 9). We found that the contacts mostly involved the T4 lysozyme (T4L) fusion domain on the extracellular part of the receptor, and to a lesser extent TMH5 and the intracellular loop (ICL) 2. The intracellular tips of TMHs 6 and 7 or ICL3 that strongly determine the active-like conformation of the receptor are not engaged in crystal packing contacts. In particular, the electron density is partially missing for ICL3, suggesting a high level of flexibility. Such properties would not be observed if crystal packing contacts were stabilizing the intracellular region of the receptor and suggest that crystal packing does not bias the receptor conformation.

The Hyd_high7 structure was determined without bound Gs, while our active-state design model included Gs docked into the G protein binding site, generating specific receptor interactions and distortions on the intracellular sides of TMH6 and TMH7 (Fig. 5f,g). Nevertheless, with the exception of M95^3.43^ directly affected by the binding of Gs through the movements of the contacting Y288^7.53^ (Fig. 5k), the conformations of the residues involved in the designed TMH interaction networks agreed with near-atomic accuracy the predicted and experimental structures (Fig. 5h,i). The root mean squared deviation (RMSD) between the experimental structure and the design model was only 1.5 Å over the entire TM region and was reduced to 1.2 Å when the two helical turns of TMH6 contacting Gs were excluded. Overall, the structural prediction of the TMH core largely validates the designed allosteric motifs.

**Extended Data Fig. 10:**
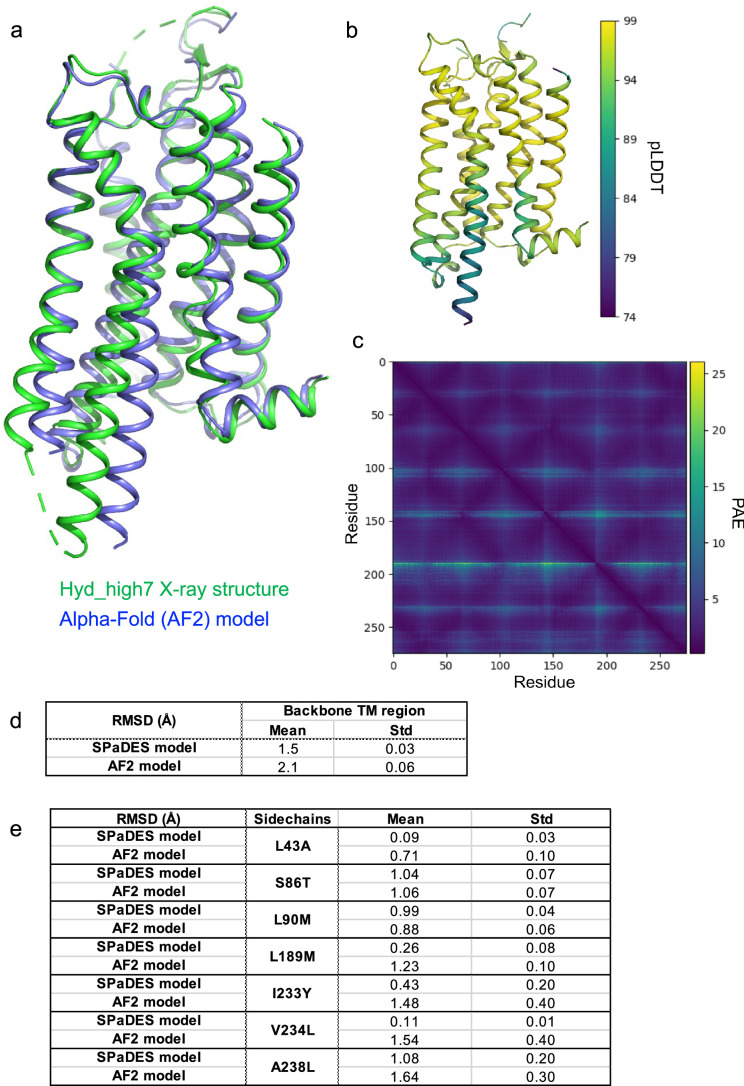
Structure prediction of the Hyd_high7 design by Alpha-Fold2. **a**, Superposition between Hyd_high7 X-ray structure and Alpha-Fold2 model (AF2). **b**, AF2 model colored according to pLDDT score. **c**, pAE plot. **d, e**, Structural differences between the models generated by SPaDES or AF2 and the Hyd_high7 X-ray structure (provided as mean ± std calculated over 10 lowest energy models). **d**, Cα RMSD over the transmembrane region. **e**, RMSD of designed residue side-chains after local alignment of surrounding 6 residues. Source data

To assess whether our approach is superior to other methods for the design of solvent-mediated protein interactions and structures, we next examined whether AlphaFold could predict the structures of our designs. As expected, AlphaFold predicted the overall fold of the Hyd_high7 design correctly as it does not particularly differ from native GPCR structures present within the training data (2.1 Å RMSD over the entire TM region, Extended Data Fig. 10). However, AlphaFold failed to accurately predict the precise structural details of side-chain conformations around solvated sites. The average side-chain RMSD for the AlphaFold models was 1.22 Å, compared with 0.57 Å for the models generated by SPaDES (Extended Data Fig. 10). These results are not surprising given that most experimental structures do not include specific solvent positions and mediated interactions. In addition, as the solvated properties (for example, polar contacts and microcavities occupied by water molecules) in our designs are quite distinct from those of native GPCRs, we would not expect AlphaFold to recapitulate them.

To directly assess how the designs rewired the solvent-mediated interaction networks defining the allosteric signal transduction pathways in receptor active states, we next compared the Hyd_high7 structure with native GPCR structures. Experimental evidence for solvent occupancy in GPCRs primarily derives from inactive-state structures owing to the fewer, often poorly resolved, number of active-state structures. However, the highest-resolution GPCR active-state X-ray structure measured so far, that of μ-opioid (mOR) bound to agonist and nanobody, was determined at a resolution of 2.1 Å and enabled direct assignment of electron densities to buried solvent molecules^26^. To compare with the mOR structure, we refined Hyd_high7 using the Phenix.SPaDES_refine software, which we developed to build and optimize protein models with explicit solvent against experimental crystallographic datasets (Methods). Phenix.SPaDES_refine significantly enhanced the quality of the Hyd_high7 structural model as MolProbity, all-atom clash and *R*-free scores improved from 2.22, 3.72 and 0.3086 to 1.42, 2.12 and 0.3240, respectively (Supplementary Table 8).

**Fig. 6:**
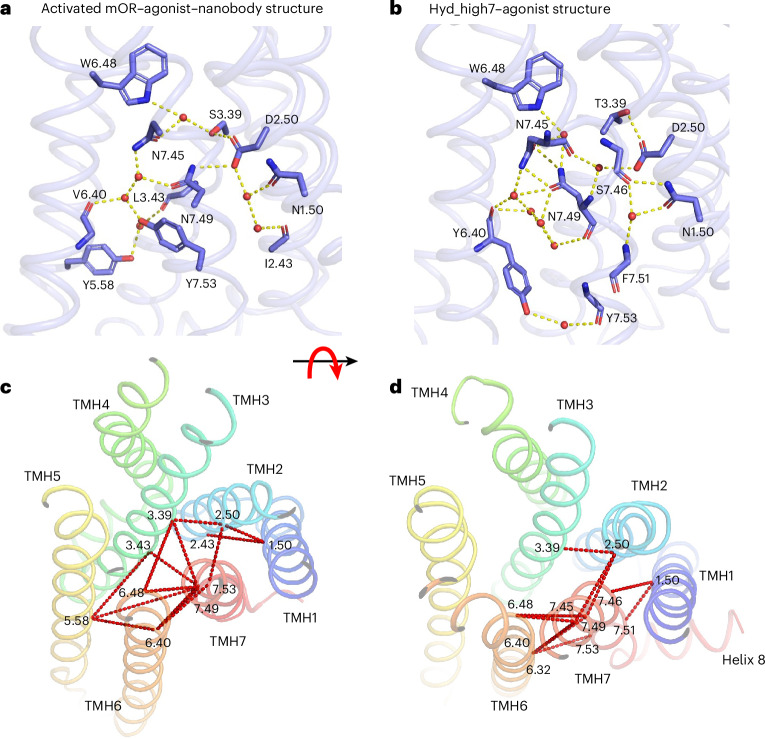
Hyd-high7 is built of a synthetic allosteric interaction network distant from those of natural GPCRs. **a**,**b**, Solvent-mediated polar interaction networks at static–switchable TMH interfaces in mOR (PDB: 5C1M) (**a**) and Hyd_high7 (**b**) active-state X-ray structures. Water molecules are represented as red spheres and water-mediated hydrogen bonds are indicated by yellow dotted lines. **c**,**d**, Interhelical (direct and water-mediated) polar interactions in the TMH core region extracted from the mOR (5c1m) (**c**) and Hyd_high7 (**d**) active-state X-ray structures viewed perpendicular to the membrane bilayer. Polar interactions are represented as dotted red lines on the backbone representation of the receptors. The designed mutations rewired the inter-TMH polar interaction network by eliminating native polar contacts between TMH3 and TMHs 5–7 while enhancing TMH7 connections with neighbouring TMHs 1, 2 and 6.

All residues interacting with solvent molecules in the mOR structure are conserved in WT A2AR, thus justifying direct comparison with the Hyd_high7 structural model. Hyd_high7 shares only 40% solvated side-chains with mOR (Supplementary Table 9), and comparing the two structures revealed striking differences in the allosteric interhelical polar contact networks (Fig. 6). The network in mOR bridged eight distinct TMH interfaces involving all of the TMHs except TMH4. In contrast, polar connections in Hyd_high7 mostly involved the designed static–switchable interfaces between TMHs 1–3 and TMHs 6 and 7, suggesting distinct allosteric mechanisms of receptor signalling (Fig. 6a–d). Sequence conservation analysis revealed that the combinations of designed residues in the Hyd_high7, 8, 9, 10 and 11 variants are unique within class A GPCRs (Supplementary Tables 10 and 11), further demonstrating that our approach can produce novel and potent solutions to solvent-mediated allosteric signalling functions.

In contrast to studies of natural GPCR sequences and structures^26,27^, our results reveal that constitutive and ligand-induced activities can be designed concurrently through a wide range of interhelical polar contact networks, providing key allosteric coupling hotspots such as between the switchable TMH6 and TMH7 in GPCRs, efficiently propagating structural change and movement.

## Discussion

Protein catalysis and allostery often rely on subtle motions and the complex interplay of protein–ligand, solvent-cooperative interactions that remain very challenging to design. In this study, we developed and applied a computational approach to engineer signalling activity in receptor scaffolds through de novo designed, solvent-mediated dynamic interaction networks at switchable TMH binding interfaces that are critical for signal transduction. We rationally designed receptors with various TMH interaction networks and measured their signalling activities. Our most stable and active variant crystallized into an unforeseen active-state conformation that closely matched the design model. Hyd_high7, with its high signalling activity and thermostability, represents a new active form in the GPCR family.

Numerous studies of native rhodopsin-class GPCRs have revealed a highly conserved network of allosteric polar residues buried in the TM region, interacting through water and ion molecules^3,11,26,28^. Alanine mutations at these sites often impair signalling, demonstrating their critical role in signal transmission^29,30^. This suggests that efficient signalling relies on the precise and conserved allosteric network topologies of polar residues and water molecules. Contrary to these findings, we engineered a library of signalling receptors with allosteric interaction networks that are different from naturally evolved receptors. The TMH and solvent interactions that mediate long-range communication pathways were considerably rewired, defining novel switchable network topologies (Figs. 2 and 6). We identified that hydration density at static–switchable TMH interfaces is a key determinant of signalling efficacy, allowing us to design receptors with diverse responses. These results, along with the unique features of Hyd_high7, highlight the plasticity of ‘wet’ polar interaction networks in promoting conformational flexibility and signal propagation. In light of these findings, it is tempting to speculate that such plasticity may enhance signal transduction robustness to sequence variations and facilitate the evolution of complex signalling responses involving multiple allosteric pathways.

Interestingly, while Hyd_high7 strongly responded to adenosine binding, it showed a reduced dynamic range (that is, ligand-induced activation) compared with the native A2AR. This might be due to the selection for high thermal stability, which could impair the conformational dynamics needed for maximal activation, or it indicates that A2AR is already close to its maximal adenosine-induced signalling capacity. Indeed, despite Hyd_high2 and Hyd_high3 achieving higher maximal activity than WT A2AR, their dynamic responses remained similar to the native receptor.

Our design calculations were guided only by the physics of protein interior solvation, microswitch structural plasticity and the stability of multiple protein conformational states. With no selection pressure for sequence conservation, our in silico design approach is agnostic to any evolutionary processes driving native protein sequences and structures. Our functional designs suggest that the interaction space mediating protein movements and allostery is far broader and largely unexplored compared with native proteins.

Protein design has typically focused on the design of highly stable structures and associations through extensive hydrophobic interactions^31^ or geometrically optimized hydrogen bond networks that ignore solvent-mediated microswitches^6,7^. Examples of designed conformationally sensitive protein switches include a two-state switching four-helical peptide bundle for Zn transport stabilized by alternating hydrophobic packing interactions^32^ and reprogramming receptor signalling via the engineering of amino acid microswitches at allosteric sites^17,33^. To the best of our knowledge, our work provides the first evidence that membrane receptors with synthetic switchable polar cores can be rationally designed through programmable solvent-mediated interaction networks.

Protein–solvent and ligand-mediated contacts are crucial for protein catalysis, binding, transport and signalling. By rationally designing these interaction networks and reprogramming long-range communication pathways, our approach assists in elucidating receptor signal transductions, protein recognition and catalytic mechanisms. It also opens up possibilities for engineering diverse novel protein functions, including binding inhibitors, enzymes, biosensors impacting cell therapies and synthetic biology. In addition, it could accelerate the crystallographic structure determination of challenging membrane proteins and the discovery of innovative drugs.

## Methods

### Gene construction and expression

Haemagglutinin (HA)-tagged *A2AR* gene in pcDNA3.1(+) was obtained from the complementary DNA library. Genes coding for designed variants were synthesized. Plasmids were transiently transfected into HEK293T cells (ATCC) using Genejuice (Novagen/EMD Millipore, 70967). HEK293T cells at 70–80% confluency in 15 cm tissue culture plates were transfected with 4 μg DNA per plate. After 24 h, the transfection medium was replaced with standard growth medium DMEM supplemented with 2 mM l-glutamine (Lonza Biosciences, BE12-604F), 100 μg ml^−1^ penicillin, 100 μg ml^−1^ streptomycin (Thermofisher, 15070063) and 10% fetal bovine serum (Corning, 35-010-CV), and the cells were grown for an additional 24 h before collection.

### Membrane preparation

Membranes were prepared from transfected cells using sucrose gradient centrifugation as previously described^17^. Briefly, cells from 10 cm plates were collected with a cell scraper using PBS solution (VWR, 45000-448). The cells were then pelleted and resuspended in cold hypotonic buffer (1 mM Tris-HCl, pH 6.8 (VWR,IC816100), 10 mM EDTA (VWR, EM-4005) and protease inhibitor cocktail (Promega, PAG6521)) and forced through a 26-gauge needle three times. The cell lysate was layered onto a 38% sucrose solution in buffer A (150 mM NaCl (Merck, S9888), 1 mM MgCl (Merck 442611-M), 10 mM EDTA, 20 mM Tris-HCl, pH 6.8, and protease inhibitor cocktail) in SW-28 ultracentrifuge tubes. The cells were centrifuged at 40,000 *g* at 4 °C for 20 min, followed by collection of the interface band with an 18-gauge needle. The collected solution was transferred to Ti-45 ultracentrifuge tubes (Beckman) and the volume brought up to 50 ml with buffer A. The sample was then spun at 185,000 *g* at 4 °C for 30 min. The membrane pellets from each 10 cm plate were resuspended in 0.5 ml buffer A and stored at −80 °C in 100 μl aliquots.

### Designed receptor partial purification

The designed variants were partially purified from thawed membrane preparations immediately before assaying via anti-HA agarose beads (Thermofisher, 26181). The membrane preparations were solubilized with 1% *n*-dodecylmaltoside (DDM; Merck, D4641) for 1 h at 4 °C and loaded onto anti-HA agarose beads for 1 h at 4 °C. The beads were washed with Tris-buffered saline (TBS) containing 0.1% DDM wash buffer three times and the HA-tagged receptor variants were eluted with HA peptide (1 mg ml^−1^ in TBS with 0.1% DDM). The purity of affinity-purified protein samples was assessed by Coomassie-stained SDS–PAGE and western blot analysis for all receptor variants.

To determine whether the bands observed in western blots resulted from glycosylation, HEK293T cells expressing WT A2AR were treated with 50 μg ml^−1^ tunicamycin (Sigma, T7765), a glycosylation inhibitor, by replacing the transfection media with culture media supplemented with tunicamycin. The cells were collected after treatment for 24 h (48 h post-transfection) as described above. Partially purified A2AR samples from untreated or treated cells were analysed by western blotting to determine the extent of glycosylation inhibition^34^ (Supplementary Fig. 1).

### Expression and purification of the G protein Gs

G-alphaS (GαS) was cloned into pFastbacI (Thermofisher, 10359016) followed by transformation into DH10α cells (Agilent, 200231). Recombinant bacmid DNA was isolated and transfected into Sf9 insect cells (Sigma, 71104-M) with Cellfectin II (Thermofisher, 10362100). The transfected cells were grown at 28 °C for 72 h followed by centrifugation in 15 ml Falcon tubes to pellet cell debris. The supernatant was saved as P1 viral stock. The virus was amplified by infecting the Sf9 cells with P1 viral stock solution at a twofold multiplicity of infection and cultured for 72 h before collection. This amplification process was repeated to generate a high-titre P3, which was used for infection and protein expression. The P3 stock was used to infect the Sf9 cells at a multiplicity of infection greater than 4, which were collected 48–60 h post infection. The cells were then washed three times in ice-cold PBS and resuspended in homogenization buffer (10 mM Tris-HCl, 25 mM NaCl, 10 mM MgCl_2_, 1 mM EGTA (Sigma, 234626), 1 mM dithiothreitol (DTT; Sigma, 10197777001), protease inhibitor cocktail and 10 μM guanosine diphosphate (Sigma, G7127), pH 8.0). GαS was purified as described previously^35^. Antibodies were purchased from Santa Cruz Biotechnology.

### G protein activation assays

Receptor variants were assayed for their ability to induce guanine nucleotide exchange in GαS. To measure constitutive activity, the reaction mixture consisted of 4 μM GαS, 20 μM [^35^S]GTPγS mix (guanosine 5′-(γ-thio)triphosphate tetralithium salt) (Revvity, NEG030H001MC), 50 mM Tris-HCl, pH 7.2, 100 mM NaCl, 4 mM MgCl_2_ and 1 mM DTT. The receptor concentrations were ~5–20 nM per sample, as estimated from the absorbance of the anti-HA agarose-purified samples at 280 nm. For all samples used in the reactions, western blotting was performed to normalize for receptor quantity using monoclonal anti-HA antibody (Thermo Scientific, 26183). The reactions were initiated by adding 150 μl partially purified receptor samples to 300 μl of the reaction mixture and incubating on ice for 1 h. To measure the ligand-induced receptor activities, a saturating concentration of adenosine (10 μM; Sigma, A9251) was added to the reaction mixture. The reactions were stopped by filter binding onto Millipore nitrocellulose filters (Merck, HAWP04700). The filters were washed three times with ice-cold TBS before incubation with scintillation fluid. Radioactivity counts were measured on a Beckman LS6000 scintillation counter. Mock transfected samples (using empty pcDNA vector) and WT receptor served as controls. Background binding, as measured in the mock transfected samples, was subtracted and the activities of the receptor variants were normalized relative to the WT receptor via densitometry analysis of western blots using ImageJ software. The statistical significance of the differences in the constitutive or ligand-induced activities was assessed by Student *t*-tests using GraphPad software. All receptor activities were obtained from at least two independent experiments and, in any given experiment, technical triplicates were recorded for each condition.

### Apparent agonist-bound receptor stability

The apparent stability of the agonist-bound designed variants was determined by measuring either receptor binding to agonist (apparent melting temperature) or receptor activities (apparent receptor half-life) as a function of temperature. To measure the apparent melting temperature, anti-HA agarose affinity-purified receptor samples prepared as described above were first pre-incubated at increasing temperatures for 30 min. The samples were then incubated with a saturating amount of [^3^H]adenosine (10 μM; Revvity, NET1161250UC) in a total volume of 150 μl per sample for 1 h on ice, followed by loading onto Millipore nitrocellulose washed for scintillation counting as mentioned above. To measure the apparent receptor half-life, purified receptor samples were pre-incubated at 37 °C for 0–60 min before addition to the reaction mixture containing GαS, [^35^S]GTPγS and 10 μM adenosine, as described above for the measurement of G protein activation. The apparent melting temperatures and active-state half-life curves were fitted using GraphPad Prism software and analysed for statistical significance by Student *t*-tests. All receptor activities were obtained from at least two independent experiments and, in any given experiment, technical triplicates were recorded for each condition.

### Modelling of receptor active-state conformation

The modelling was performed before the release of the A2AR active-state structure bound to mini-Gs (ref. ^22^). Hence, we modelled the receptor active conformation by homology to the fully active Gs-bound B2AR structure using the method IPHoLD^14^^,18^. As the sequences of B2AR and A2AR on the extracellular side diverge substantially, we took advantage of available partially active agonist-bound A2AR structures. We grafted the extracellular region of the UK-432097 agonist-bound A2AR structure^36^ onto the intracellular region of the fully active homologue Gs-bound (B2AR) X-ray structure to create a chimeric homologue template for the fully active conformation of the agonist and Gs-bound receptor template. Intracellular loop regions were rebuilt de novo in the presence of bound Gs as described previously^37^, and the entire structure was relaxed using SPaDES, a method developed for modeling membrane proteins with explicit solvent^15^. Around 20,000 independent simulations were performed and, from those, the 10% lowest-energy models were clustered, with the centres of the most populated clusters selected as fully active-state receptor templates for the design calculations of activating microswitches and solvent-mediated networks. The A2AR inactive-state X-ray structure bound to the high-affinity antagonist ZM241385 (ref. ^38^) was selected as a representative inactive-state model for the receptor design templates.

### Computational design of activating microswitches and solvent-mediated networks

The inactive- and active-state templates described above were used as starting models in the design calculations of interaction networks bridging the receptor TMHs. Receptor residues in the extracellular and intracellular binding sites were taken from the native adenosine receptor A2AR to preserve adenosine ligand and G-protein binding. We focused our design calculations on the interface positions between the static TMHs 1, 2 and 3 and the switchable TMHs 6 and 7 and selected a total of 22 designable positions.

Owing to the substantial computational overhead associated with the side-chain and solvent rotamer repacking in SPaDES, we restricted the sequence search space to hydrophobic (that is, Leu, Ile, Met, Val and Ala), uncharged polar (that is, Ser, Thr, Asn and Gln) and small charged residues (that is, Asp and Glu). We included Tyr in the polar group for the design space at position 6.48 as the distances between TMHs 6 and 7 at that site are greater and could fit large hydrophobic residues.

We performed the design selection in two steps. In the first step, sequences were designed to either increase or decrease the energy of the active state. In the second step, selected sequences were then threaded and extensively relaxed in both the active- and inactive-state models with explicit hydration to calculate the impact of the designed mutations on that state. The energy difference between both states relative to A2AR defines the quantity ΔΔ*G*_apo_, which is a measure of the impact of the design on the conformational equilibrium of the receptor and the relative conformational stability of each state. Specifically, the design protocol uses a simulated annealing Metropolis Monte Carlo minimization algorithm to sample sequence space. The energy of each sequence and solvent-mediated interaction network is calculated using a protein side-chain and solvent molecule rotamer repacking protocol, followed by minimization of the receptor structure over all the conformational degrees of freedom using the SPaDES energy function. In these calculations, sites interfacing the static and switchable TMH helices were defined as hydratable and repackable, while residues within 5 Å of hydratable sites were set as repackable. We used the SPaDES hydrate water12 scoring function with pre-Talaris2013 behaviour and initialized the membrane. In the first design step, the active-state energy of each design was assessed from 50 independent models and used to select designs for the second design step. In that stage, the active and inactive states of the selected designs underwent extensive relaxation using SPaDES to provide an accurate estimation of the free energies of the active (Δ*G*_active_) and inactive (Δ*G*_inactive_) states as well as ΔΔ*G*_apo_. Specifically, we generated 500 independent models for each identified design in each state. We found excellent convergence in the top ten scoring models (the average standard errors in Δ*G*_inactive_ and Δ*G*_active_ were 0.32 and 0.21 REU, respectively) and subsequently selected the best scoring model for structural analysis in each case.

We found that the most frequent impact of mutations in the receptor core was the destabilization of both the inactive- and active-state structures (positive Δ*G*_inactive_ and Δ*G*_active_). As this would probably lead to protein unfolding, such sequences were systematically discarded during our calculations before we applied the selection criteria 1–3 described below. Typical Δ*G* thresholds from WT A2AR for unselected designs were >8 REU for Δ*G*_inactive_ and Δ*G*_active_.

Overall, nine single- or double-point mutants and five combinatorial designs, including thirteen distinct designed residues, were selected for experimental validation. These designs were selected because they sampled different levels of the following three criteria: (1) conformational stability of the active state compared with that of the inactive state, (2) water-mediated hydrogen bond connectivity between static and switchable TMHs, and (3) the strength of protein–ion interactions locking and stabilizing the protein into a specific conformation.

Unselected designs displayed the following trends in criteria values:

1. Criterion 1: ΔΔ*G*_apo_ (that is, Δ*G*_active_ − Δ*G*_inactive_) was often higher than 5 REU and would likely correspond to a loss in both basal and ligand-induced signalling activities.
2. Criterion 2: we often observed a loss of the water-mediated hydrogen bond connectivity between static and switchable TMHs that mirrors the properties of the Hyd_low designs selected to validate our design method. The typical threshold for criterion 2 (that is, the number of static–switchable water-mediated hydrogen bonds) for unselected designs was <18.
3. Criterion 3: we did not observe any striking specific trend for the ion interaction energies that would be systematically orthogonal to criterion 2. If a mutation occluded the Na^+^ binding site preventing ion binding, it also often led to a loss in water-mediated interactions. As reflected by the distribution of the mutational effects on Na^+^ interaction energies, criterion 3 is a second-order effect that is not as discriminative as criteria 1 and 2 for selecting competent signalling receptors.

An example script to run SPaDES hydrate is provided in the Supplementary Methods. All designed final models, scripts to regenerate trajectories for the results presented in this report, documentation and detailed demos are available via GitHub at https://github.com/barth-lab/SPaDES_DESIGNS.

### Relationships between basal activities and the equilibrium between ligand-free states

The allosteric two-state model (ATSM) provides a theoretical framework for relating the calculated thermodynamic properties of a receptor to its measured allosteric activity. According to the ATSM, we can expect a quasi-linear relationship between basal (that is, ligand-free) activity and free energy in our study. Without ligand stimulus, the basal activity (BA) is directly related to the fraction of ligand-free receptor R in the active state, which can be calculated from the equilibrium constant of the ligand-free receptor in the inactive and active state (*K*_R_) and therefore from the free-energy difference between the two states (Δ*G*_apo_) by solving the following equations:

1 $${\rm{BA}}={K}_{{\rm{R}}}/(1+{K}_{{\rm{R}}})$$

2 $${\Delta G}_{{\rm{apo}}}=-RT\,\mathrm{ln}[{\rm{BA}}/(1-{\rm{BA}})]$$

Reliably fitting the Boltzmann distribution described by equation (2), where *R* is the molar gas constant and *T* is the temperature, would require a dataset covering the complete range of basal activities (for example, from <5% to >95% of the maximal receptor activity). Nevertheless, the relationship between BA and ΔΔ*G*_apo_ is quasi-linear within the range of measured receptor basal activities in our study (Supplementary Fig. 3). To a first approximation, such linear relationships enable a direct quantitative comparison between experimental basal activity and ΔΔ*G*_apo_ energy that is independent of the energy units. The linear coefficients relating basal activities to Rosetta-based energy units or kilocalories per mole are different because the former differ from the physical free-energy units.

### Topologies of water-mediated interaction networks

Protein–solvent interactions were analysed as a network through graph analysis using the software Cytoscape^39^. In this analysis, each pair of interacting residue and water molecule was assigned a pair of nodes, and each hydrogen bond interaction between them represented by an edge. For each designed protein in each state, solvent positions were extracted from the 10% lowest-energy structural models and clustered to identify positions common to multiple models. These cluster centres defined the input solvent nodes for Cytoscape. A water molecule mediating a static–switchable interaction was defined as a water node sharing at least one edge with a residue node on a static TMH and sharing at least one edge with a residue node on a switchable TMH. A water node connected to a residue node on a static TMH can also mediate a static–switchable interaction if it is sharing an edge with another water node connected to a residue node on a switchable TMH and vice versa. Each edge of these types defined a static–switchable water-mediated interaction in our analysis.

### Modelling sodium binding

We expanded SPaDES to model buried ion molecules in protein structures. As SPaDES was developed for protein design calculations that require joined sampling of large structure and sequence spaces, it relies on a compromise between accuracy and speed. The approach approximates ion solvation energies by the interaction energy between the ion and a shell of water molecules (in the bulk) or a combination of water molecules and protein residues in direct contact with the ion (in the protein core). SPaDES energies are thus dominated by short-range interactions and neglect configurational entropic effects or long-range interactions. However, as the design goal was to identify amino acid substitutions that impact the direct contacts with ion molecules, the approximations underlying the model are valid and capture the primary enthalpic determinants of Na^+^ interactions in protein cores.

Specifically, the method models the effects of amino acid substitutions on protein–ion interactions in the vicinity of known ion binding sites and involves the following steps:

1. An ion is placed at the origin of a voxelated mesh grid at 0.5 Å intervals in the *x*, *y* and *z* directions in a 5 Å box around the known ion binding site obtained from the input structure. All possible positions of the ion are sampled exhaustively in this grid by moving from −2.5 Å to 2.5 Å in each Cartesian direction, creating a total of 1,331 initial ‘ion placement’ states.
2. The ion-placed protein structures are hydrated and repacked using SPaDES and clustered using a hierarchical tree based on the geometric position of the ion and its distance matrix from surrounding residues. The top ten scoring (by energy) clusters are selected for ion placement refinement.
3. In each of the selected structures, the ion is replaced in a voxelated mesh grid at 0.05 Å intervals from −0.2 Å to 0.2 Å in the current ion binding site. This results in 7,290 starting structures for the next step.
4. These ion-placed protein structures are hydrated and repacked using the SPaDES method and the top ten scoring structures selected as the final poses for analysis.

Our simulations converge to highly reproducible Na^+^ interaction energies as the average standard error lies within 0.016 REU (Supplementary Table 3).

Scripts to reproduce the ion sampling results, including a detailed demo, are available via GitHub at https://github.com/barth-lab/SPaDES_DESIGNS.

The ion occupancy in a specific state of the receptor was derived by calculating the Boltzmann distribution of an ion molecule buried in the receptor versus fully solvated in the bulk. Energies of Na^+^ in the bulk were calculated for a Na^+^ ion fully solvated in a 125 Å^3^ cubic grid. Briefly, Na^+^ was placed at the centre of a cubic grid, solvated and then repacked using SPaDES to identify the optimal network of ion–water interactions. Overall, 500 individual trajectories were run, with the lowest-energy being a consistent outcome with six water molecules packed around the ion. The ion–water interaction energy of the lowest-energy configuration was taken as the ion energy in the bulk and used to calculate the Boltzmann distribution. The ion occupancy was directly calculated from the Boltzmann factors of each state.

Deriving Boltzmann occupancies from SPaDES energies required the translation of SPaDES energy units into a thermodynamic scale (that is, kilocalories per mole). We found that the relative effects of the designed mutations on Na^+^ occupancy were similar for scaling factors translating SPaDES energies into thermodynamic quantities that ranged from 1 REU = 1 kcal mol^−1^ to 1 REU = 3 kcal mol^−1^, covering a range of Rosetta forcefields. Overall, these results indicate that, at the qualitative level, our conclusions on the impact of the designs on receptor–Na^+^ binding interactions are valid and robust to the scoring function.

### Sequence conservation analysis

In assessing sequence conservation, we first performed a sequence alignment over all class A GPCRs over the structurally conserved TMHs 1–7 via the GPCRdb database^40^. We next performed a Basic Local Alignment Search Tool (BLAST) search over this multisequence alignment to calculate *E* values with respect to human A2AR (where an *E* value of 10 indicates 10 hits would be expected to be found by chance given the same size of a random sequence database). We selected all those sequences with an *E* value smaller than 10^−2^ to account for distant homologues when assessing the conservation of design and solvated sites. At each design site (1.42, 2.46, 2.50, 3.39, 3.43, 5.55, 6.40, 6.41, 6.45 and 7.49) and additional adjacent solvated sites (1.50, 2.43, 5.58, 6.48, 7.45, 7.51 and 7.53), we calculated both the residue conservation with respect to A2AR (or mOR in the case of solvation) and the occurrence of the designed amino acid. The motif analysis reports on how frequently the combinations of mutations selected in the designed receptors occur in natural receptors. Specifically, we searched through all class A GPCRs for occurrences where at least two mutations, such as Asn2.50 and Asp7.49, corresponding to Hyd_High2, naturally occur together. Combinations of mutations not reported in Supplementary Table 11 were not observed.

### Structure prediction using AlphaFold

AlphaFold2 was used to predict the structures of A2AR and Hyd_high7 without bound G protein^41^. AlphaFold2 Multimer^42,43^ was also applied, wherein the auxiliary sequence of a bound G protein was used to steer template generation. The best Amber-refined output models had an RMSD of 0.44 Å and 0.85 Å for regions with a predicted local distance difference test score >80 for the inactive-state (PDB: 8GNE) and active-state (PDB: 6GDG) structures of A2AR.

### Expression vector design of the T4L fusion construct

There are numerous, successful examples of T4L functioning as a stabilizing chaperone protein for numerous GPCR proteins deposited in the PDB. Our strategy was to follow those successful examples and fuse a cysteine-free T4L (ref. ^44^) to the N terminus of A2AR with one exception: we decided to use a T4Lcp designed by Cellitti et al. that relocates the N-terminal A helix to the carboxy terminus, creating subdomains that are contiguous in sequence and linked by a G4S Poly-Glycine-Serine sequence^45^. T4Lcp has been crystallized at a higher resolution (1.8 Å) than other T4L constructs and may therefore have a higher probability of positively impacting SPaDES crystallization. The T4Lcp SPaDES fusion sequence was optimized using the structure of the T4Lcp protein (PDB: 2O7A) as follows. To link the two proteins, we designed small linkers between the C terminus of T4Lcp and the N-terminal amino acid of the Hyd_high variants. Following in silico screening and experimental testing, the two-amino acid linker Ala-Pro was found to be the most successful construct that resulted in a stable, soluble protein and, foremost, the one that crystallized.

### Cloning and Baculovirus expression of T4Lcp–Hyd_high variants

A T4Lcp 207A–Hyd_high7 fusion including A2AR encompassing residues 2–316 with designed mutations L48A, S91T, I238Y, L194M, V239L, A243L and L95M (numbered relative to reference sequence NP_000666) was PCR-amplified and topoisomerase-cloned into a custom topoisomerase-adapted pFastBac (KX) vector (Life Technologies). The cDNA from this clone was named 14022b11KXem1h1 and its sequence was verified. Expression of this vector generated MKTIIALSYI FCLVFADYKD DDDGAP/ T4L 207A/ AP/A2AR aa2–316 plus L48A, S91T, I238Y, L194M, V239L, A243L and L95M /GS/LVPRGS/HHHHHHHHHH. T4Lcp 207A corresponds to amino acids 2–124 (numbered relative to reference sequence NP_049736). Standard Baculovirus expression using a modified version of the Bac-to-Bac system protocol (Life Technologies, 10359016) in combination with the DH10EMBacY bacmid (Geneva Boiotech, DH10EMBacY, 12 × 100 µl) was used to generate the virus. T4Lcp–A2AR constructs were expressed in Sf9 cells, which were cultured for 48 h, collected by centrifugation and pelleted for storage at −80 °C before purification.

### T4Lcp–Hyd_high7 purification

Protein purifications were carried out using cobalt affinity, thrombin cleavage, PNGase F deglycosylation and size-exclusion chromatography (SEC). Infected Sf9 cell pellets were lysed in 20 mM Tris, pH 7.5, 500 mM NaCl, 0.2 mM lauryl maltose neopentyl glycol (LMNG; Anatrace, NG310) and 1 mM CGS21680 (Sigma-Aldrich). Membrane samples were collected by spinning at 44,000 r.p.m. for 45 min. T4Lcp–Hyd_high7 was extracted from Sf9 membranes with a Dounce homogenizer in a solubilization buffer comprising 20 mM DDM (Anatrace, D310), 4 mM cholesteryl hemisuccinate Tris salt (CHS; Anatrace, CH210), 50 mM HEPES, pH 7.8, 500 mM NaCl and 25 μM Cmpd-1. Talon resin (Takara, 635653) was added and mixed continuously overnight at 4 °C. The Talon resin was collected by spinning and washed extensively with Ni washing buffer comprising 5 mM DDM, 1 mM CHS, 50 mM HEPES, pH 7.8, 500 mM NaCl and 10 mM imidazole. This was followed by a second washing step using 2 mM LMNG instead of DDM, 1 mM CHS, 50 mM HEPES, pH 7.8, 500 mM NaCl and 10 mM imidazole. The protein was eluted from the resin using elution buffer comprising 250 mM imidazole, 1 mM LMNG, 50 mM HEPES, pH 7.8, and 500 mM NaCl. A desalting column was then used to exchange buffer into 50 mM HEPES, pH 7.8, 500 mM NaCl, 0.5 mM LMNG and 0.1 mM CGS21680, followed by cleavage with 20 μl thrombin and 20 μl PNGase F overnight at 4 °C. The elution sample was concentrated and loaded onto a SEC column (Superdex 200) with a buffer comprising 0.2 mM LMNG, 20 mM HEPES, pH 7.5, 500 mM NaCl and 25 μM CGS21680. The major peak fractions were combined and concentrated for crystallization and the ligand was added to a final concentration of 500 μM.

### Fluorescent thermal stability assay of T4Lcp–Hyd_high7 constructs

The receptors were purified as described for crystallization with the reducing agent removed in the final chromatography step. The receptors were diluted to 2 μM in PBS, 0.5 mM DDM, 0.1 mM CHS, 20 μM 7-diethylamino-3-(4′-maleimidylphenyl)-4-methylcoumarin (Invitrogen) and 100 μM ligand compound. Samples were incubated on ice for 1 h before measurement. Melts were completed in a Qiagen Rotor-Gene Q apparatus, monitoring the blue channel with a thermal ramp of 30–90 °C measuring the fluorescence three times per degree. The data were processed using Excel. The raw fluorescence was normalized and the melting temperatures were calculated from these data.

### T4Lcp–Hyd_high7 crystallization

T4Lcp–Hyd_high7 protein (50 mg ml^−1^) with 0.5 mM of the ligand agonist CGS21680 was mixed with monoolein containing 10% cholesterol in a protein/lipid ratio of 1:1.5 (v/v) using the twin-syringe mixing method^46^. We performed extensive lipidic cubic phase (LCP) crystallization trials on T4Lcp–Hyd_high7 using a Mosquito robot (TTP Labtech) at room temperature. Initial crystals were obtained from 0.1 M HEPES, pH 7–8, 25–30% polyethylene glycol 300 (PEG300) and 50–200 mM sodium potassium tartrate 2 days after the LCP trays were set up. Optimization screens were performed with well buffer (0.1 M HEPES, pH 7, 27% PEG300 and 50 mM sodium potassium tartrate) with 10% detergent screen (Hampton Research). Final crystal datasets were collected at a resolution of 3.9 Å using 0.1 M HEPES, pH 7, 27% PEG300 and 50 mM sodium potassium tartrate with 5 mM CYMAL-5 (Hampton Research).

### Data collection, processing and structure determination

A complete X-ray dataset was collected from a single rod-shaped crystal (~10 × 100 µm^2^) at 100 K in a single sweep of 900 × 0.2° oscillations and exposure time of 0.4 s at the DLS-I04-1 beamline at the Harwell Diamond Light Source, UK. The whole sample (that is, the LCP sample/blob in the MiTeGen loop that contained the crystal) was exposed to the beam during data collection. The diffraction data were integrated using autoPROC/XDS (refs. ^47,48^) and merged and scaled in SCALA (ref. ^49^) in the CCP4 suite^50^. The crystal structure of the T4Lcp–Hyd_high7–CGS21680 complex was determined by molecular replacement using the previously solved T4Lcp and A2AR structures, 2o79 (ref. ^45^) and modified 5IU4 (ref. ^51^), as separate search models. Clear density for the ligand was observed immediately. After numerous refinement cycles with REFMAC5 (ref. ^52^), the model was further refined using Phenix.SPaDES_refine as described below to reach reasonable *R* factors and MolProbity scores^53,34^. The final structure has been deposited in the PDB (8UGW). The solved molecular structure with explicit water molecules is available via GitHub at https://github.com/barth-lab/phenix_with_spades/tree/main/crystal_structure under the name 8UGW_with_SPaDES_waters.pdb.

### Crystal packing contact analysis

Packing contacts were estimated by calculating the buried area of the unit cell with cell mates using the Molecular Operating Environment (version 2020.01) software (Chemical Computing Group). Residues from distinct molecules related by symmetry to atoms within 4.5 Å of each other were defined as crystal contacts.

### Structure refinement with Phenix.SPaDES_refine

Structure refinement with Phenix alone or SPaDES alone resulted in either suboptimal structure geometry (as measured by MolProbity and all-atom clash scores) or suboptimal agreement with the experimental electron-density map (as measured by *R*-free), respectively. To address these issues, we considered the Phenix.rosetta_refine protocol^53^, which aims to improve the refinement of protein structures against low-resolution data by combining the strength of Phenix and Rosetta. Briefly, a RosettaScripts protocol calls Phenix routines to calculate electron-density maps such that the energy can be weighted against the crystallographic likelihood function by normalizing the gradients, while the Rosetta protocol optimizes the model geometry (repack and minimization routines). The method alternates between real-space and reciprocal-space refinement, where the Rosetta constrains reciprocal-space refinement to physically plausible conformations (which improves MolProbity scores), while the density maps restrain the Rosetta sampling in real space (which improves *R*-free).

Phenix.rosetta_refine was developed to refine water-soluble protein structures using an implicit solvent model. To apply this protocol to the refinement of membrane protein structures with explicitly modelled small solvent molecules, we implemented RosettaMembrane with SPaDES in Phenix.rosetta_refine. SPaDES builds de novo protein–solvent interaction networks and substantially improves the prediction of side-chain conformations and solvent-mediated interactions compared with standard Rosetta and other force fields. The implicit solvation model of Rosetta was replaced with the hybrid implicit–explicit solvation model of SPaDES and the protocol was modified to consider de novo modelled solvent molecules during the geometry optimization routines (repack and minimization). The weights for the real-space restraints were also optimized for the new protocol, which we refer to as Phenix.SPaDES_refine. Specifically, the Phenix.SPaDES_refine protocol performs ten separate cycles of geometry optimization: (1) each of the first three refinement cycles consists of a repack with the following scoring function weights: fa_rep = 0.22, fa_intra_rep = 0.001 and elec_dens_fast = 12.0, then a minimization with the default SPaDES scoring function weights; (2) each of the next five cycles consists of a repack with elec_dens_fast = 12.0 and a minimization with elec_dens_fast = 12.0, then a minimization with the default SPaDES scoring function weights; and (3) each of the final two cycles consists of a repack with elec_dens_fast = 12.0, then a minimization with default SPaDES scoring function weights.

We judged and reported the quality of the resulting models using various metrics, including *R*-free, MolProbity, all-atom clash score, rotamer geometry, Ramachandran plot and structural quality, as measured by Rosetta in Rosetta energy units. To assess the impact of the explicit solvent molecules in the refinement, we compared the structural models refined using the Phenix.SPaDES_refine and Phenix.rosetta_refine protocols (Supplementary Table 8). Overall, Phenix–SPaDES performed slightly better than Phenix–Rosetta. Unlike the Phenix–SPaDES model, the structure refined using Phenix–Rosetta displayed Ramachandran plot and rotamer outliers. The all-atom clash score and *R*-free were also considerably higher for the Phenix–Rosetta model, suggesting that Phenix–SPaDES was able to generate a higher model quality than Phenix–Rosetta.

Installation instructions for Phenix.rosetta_refine with SPaDES and detailed documentation and a demo for the refinement described above are available via GitHub at https://github.com/barth-lab/phenix_with_spades.

### Reporting summary

Further information on research design is available in the Nature Portfolio Reporting Summary linked to this article.

## Online content

Any methods, additional references, Nature Portfolio reporting summaries, source data, extended data, supplementary information, acknowledgements, peer review information; details of author contributions and competing interests; and statements of data and code availability are available at 10.1038/s41557-024-01719-2.

## Supplementary information

Supplementary Information Supplementary Figs. 1–9, Discussion, Tables 1–11, Data and Methods.

Reporting Summary

## Source data

Source Data Fig. 2 Statistical source data for Fig. 2b,e.

Source Data Fig. 3 Statistical source data for Fig. 3a–d.

Source Data Extended Data Fig. 9 Statistical source data.

Source Data Extended Data Fig. 10 Statistical source data.

## Data Availability

The data that support the findings of this study, including atomic coordinates, are available via GitHub at https://github.com/barth-lab/SPaDES_DESIGNS and https://github.com/barth-lab/phenix_with_spades/tree/main/crystal_structure and via Zenodo at 10.5281/zenodo.13897470 (ref. ^54^). The T4Lcp–Hyd_high7 X-ray structure has been released in the PDB (8UGW). Source data are provided with this paper.

## Code availability

The code and demo for the SPaDES protocol used in the design of the Hyd variants are available via GitHub at https://github.com/barth-lab/SPaDES_DESIGNS. The code and demo for the protocol Phenix.SPaDES_refine developed in this study are available via GitHub at https://github.com/barth-lab/phenix_with_spades.
