# Supplementary material for: Computational design of highly signalling-active membrane receptors through solvent-mediated allosteric networks
Source: Nat Chem. 2025 Jan 23;17(3):429–38. doi: 10.1038/s41557-024-01719-2 (PMC11882447; doi:10.1038/s41557-024-01719-2)
Supplement: Supplementary file 1 — Supplementary Figs. 1–9, Discussion, Tables 1–11, Data and Methods. [file 41557_2024_1719_MOESM1_ESM.pdf]

nature chemistry

Supplementary information

https://doi.org/10.1038/s41557-024-01719-2

Computational design of highly signalling-active membrane receptors through solvent-mediated allosteric networks

In the format provided by the authors and unedited

Supplementary Information table of content

Supplementary Figures 1-9

Supplementary Tables 1-11

Supplementary Discussion

Supplementary Data

Supplementary Methods

Supplementary Figure 1. Western blots of partially purified receptors using monoclonal anti-HA antibody. a-f. WT, lowX and highX designate the WT A2AR, designed Hyd_lowX and designed Hyd_highX receptors, respectively. N/A refer to other samples irrelevant for this study. The receptor MW is ~47 kDa for the monomeric form. Two bands for the receptor monomers are observed between 40 and 50 kDa and likely correspond to distinct levels of glycosylation[53]. **f.** Treatment with tunicamycin (glycosylation inhibitor) shifts the relative intensities of the high (h) and low (l) MW monomeric bands (right) but does not impact ligand induced receptor activation (left). Mean and standard deviation of 3 independent experiments are shown.

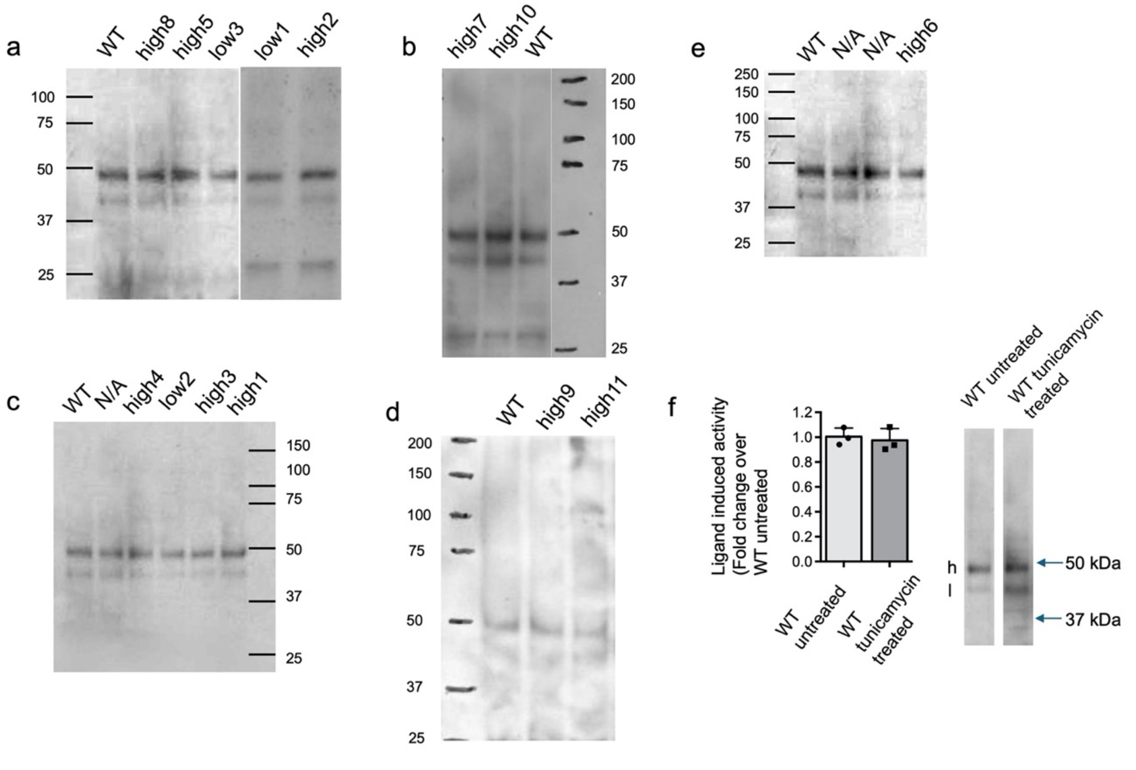

Supplementary Figure 2. Agonist titration of receptor activity. a-c. %GTPγS binding activity of partially purified designed receptors upon adenosine ligand titration normalized to fully ligand-induced activity of WT A2AR. Mean and standard deviation of 3 independent experiments are represented.

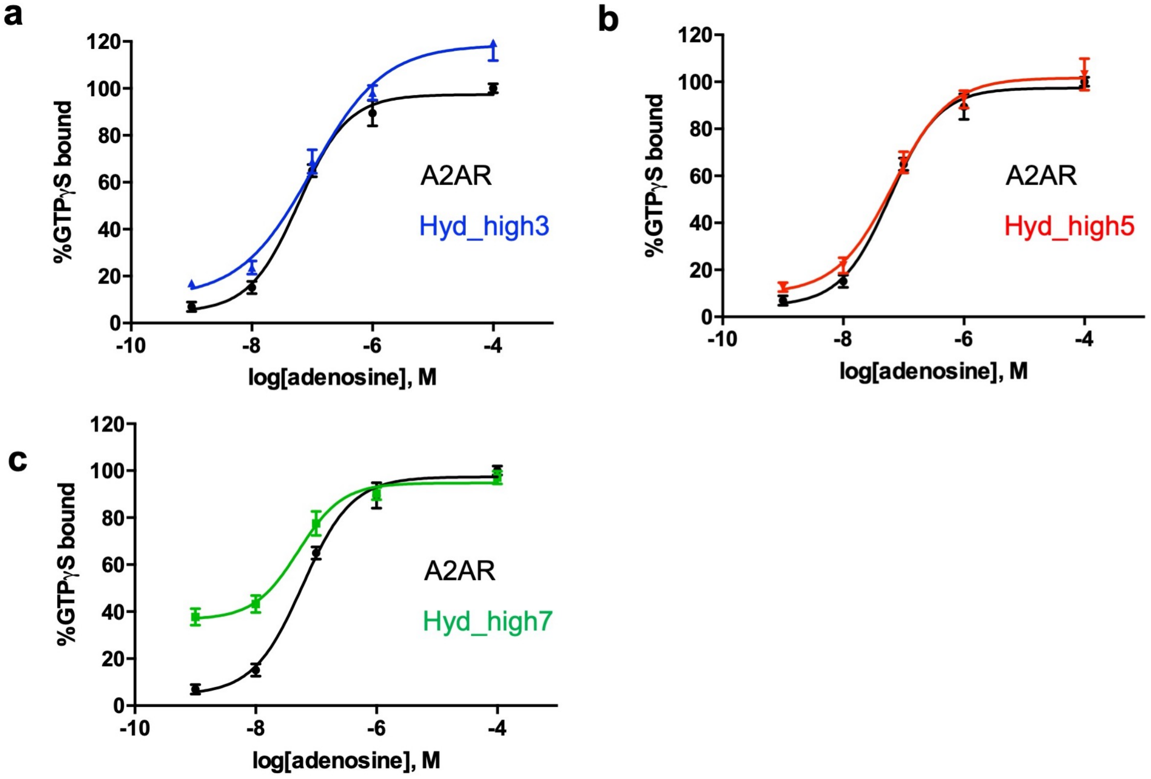

Supplementary Figure 3. Theoretical relationships between apo equilibrium shifts and basal activities according to the allosteric two state model. a. Theoretical relationship between the relative stability of the ligand-free active state versus the ligand-free inactive states (ΔG_{apo} in kcal/mol) and basal activities (represented as ratios of the maximal receptor activity) calculated by equation 2 (Methods). b. Quasilinear relationship between both quantities for the range of basal activities measured for the designed receptors (Pearson correlation coefficient r = 0.99; n=11, two-sided F test p-value<0.0001).

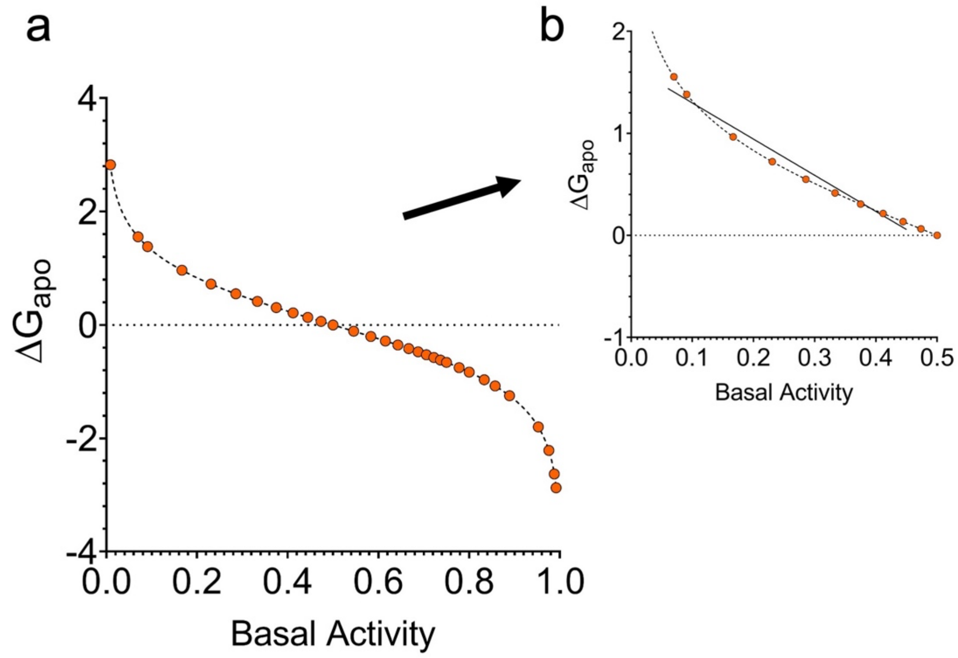

Supplementary Figure 4. Calculated sodium ion occupancy in A2AR and designed receptors. The difference in Na+ occupancy from WT is reported in blue and is calculated over the inactive, agonist-bound (partially active) and fully active states. The difference in preferential inactive state Na+ occupancy from WT is reported in red and calculated as the ratio between the occupancy of the inactive versus that of both agonist-bound and fully active states. The sodium ion occupancy in each receptor conformational state is calculated from the Boltzmann distribution of the ion in the bulk versus the lowest energy position in the protein (see Methods).

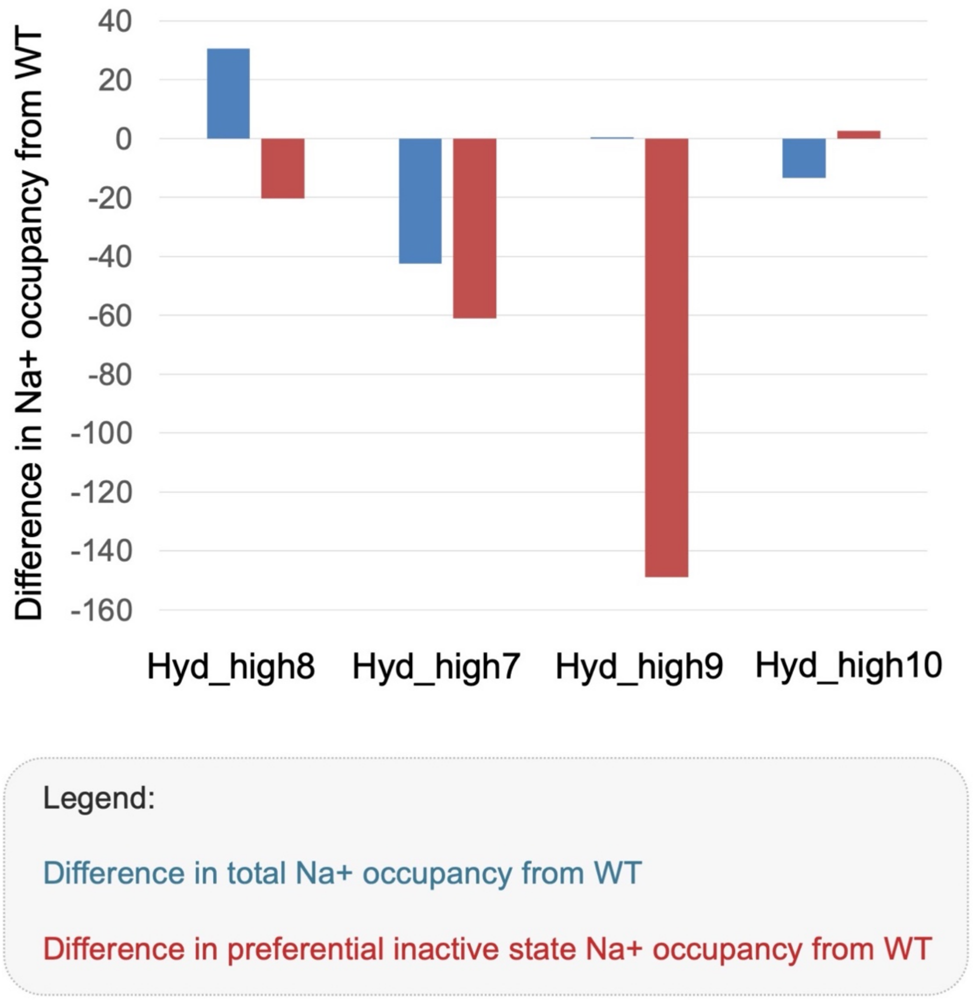

Supplementary Figure 5. Thermostability of T4L- designed Hyd_high constructs. All values correspond to the difference in melting temperature (Tm) between the designated ligand agonist-bound Hyd_high constructs and the reference T4L- A2 WT construct in the ligand-free state (i.e. APO).

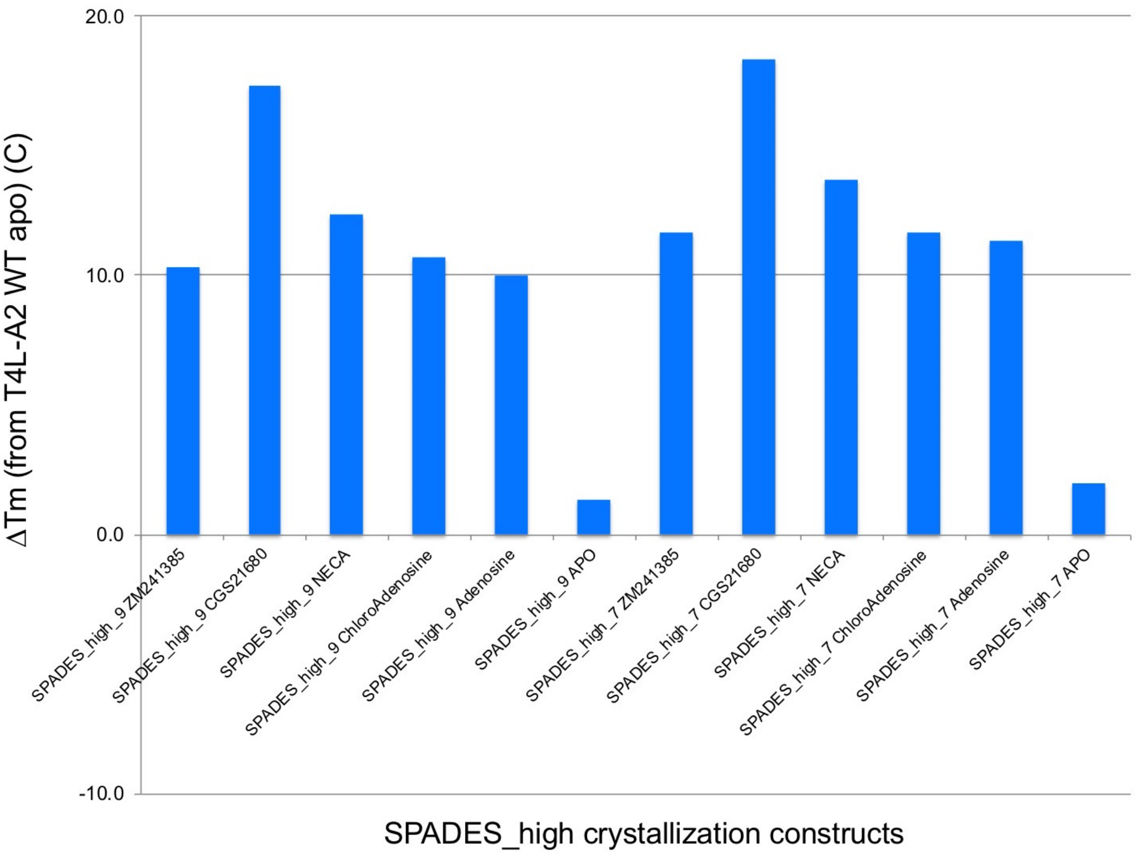

Supplementary Figure 6. Characterization and crystallization of T4L- designed Hyd_high constructs. a. Gel filtration chromatography. B12 to C5 were combined and concentrated for crystallization. **b.** SDS page gel of T4L-Hyd_high7 sample preparation. (M) molecular weight markers; (1) Ni^{2+}-NTA column flow through (1:10 dilution); (2) Ni^{2+}-NTA column wash with 10 mM imidazole buffer; (3) Ni^{2+}-NTA column wash with 2mM MNGC-12 instead of DDM with 10 mM imidazole ; (4-6) Ni^{2+}-NTA column eluate; (7) sample after cleavage overnight after Thrombin and PNGase; (8-12) elution peak (B12, C1-C4) after gel filtration. **c.** UV imaged crystals after one week with LCP screen in condition (0.1M Hepes pH 7, 27% PEG300, 50mM sodium potassium tartrate with 5mM CYMAL-5).

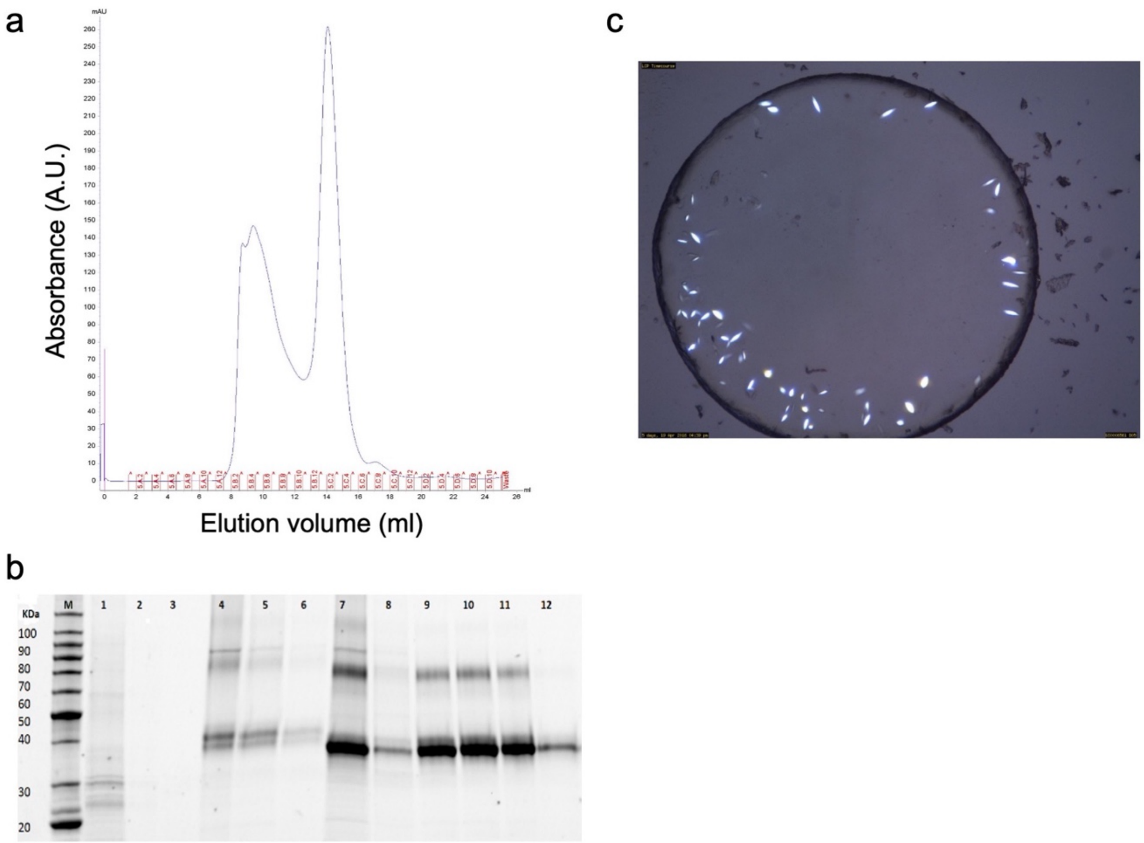

Supplementary Figure 7. Atomic resolution Hyd_high7 model in the X-ray density map. The X-ray density map and the designed Hyd_high7 model obtained after refinement using the Phenix.SPaDES_refine software are shown for the following designed residues: a. Ala48$^{2.46}$; b. Thr91$^{3.39}$; c. Met95$^{3.43}$; d. Met194$^{5.55}$; e. Tyr238$^{6.40}$; f. Leu239$^{6.41}$; g. Leu243$^{6.45}$.

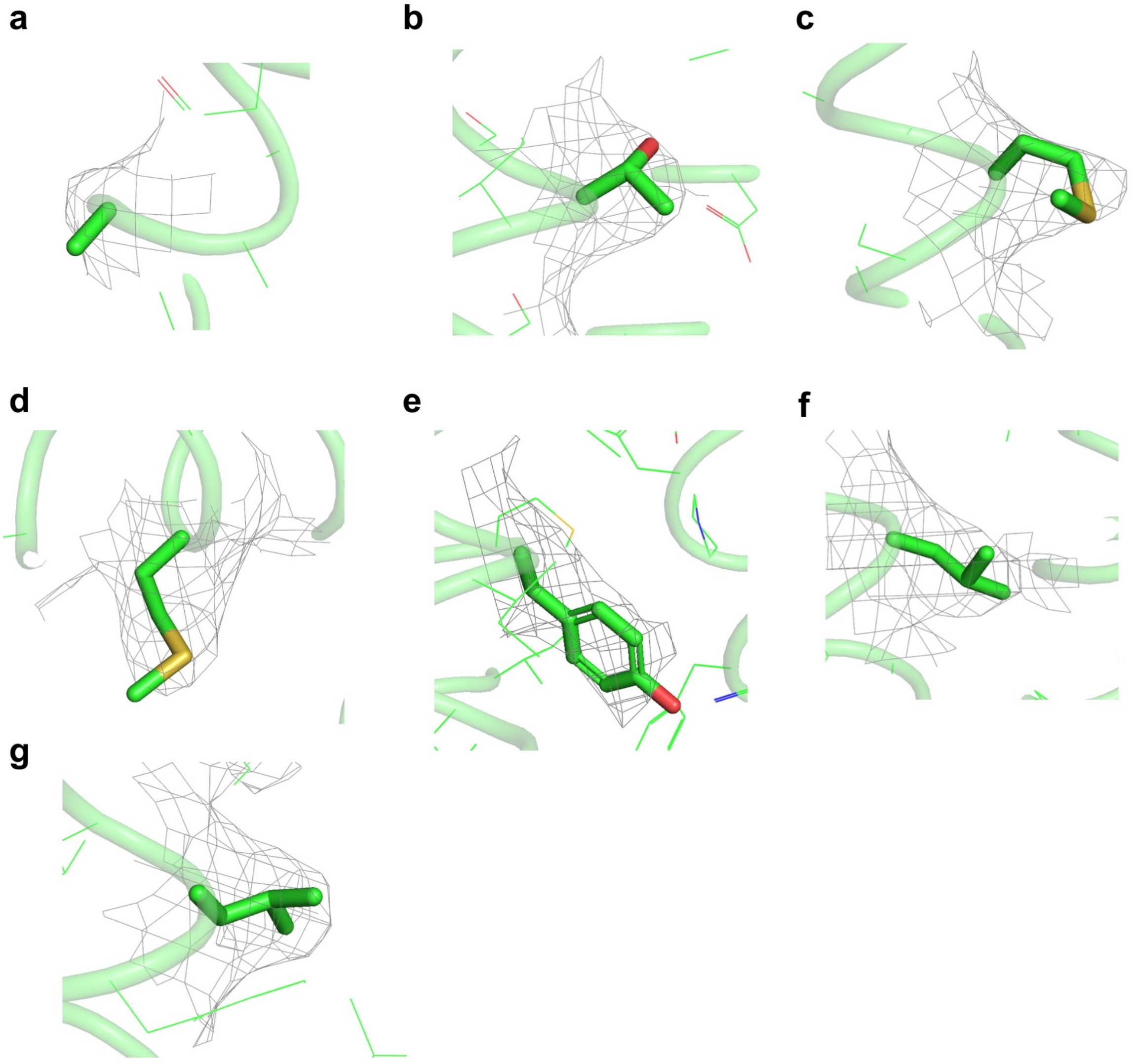

Supplementary Figure 8. Atomic resolution Hyd_high7 model in the X-ray density map. The X-ray density map and the designed Hyd_high7 model obtained after refinement using the Phenix.SPaDES_refine software are shown for the following conserved native residue microswitches that are key for G-protein binding and were introduced into Hyd_high7: a. R3.50; b. Y5.58; c. N7.49; d. Y7.53.

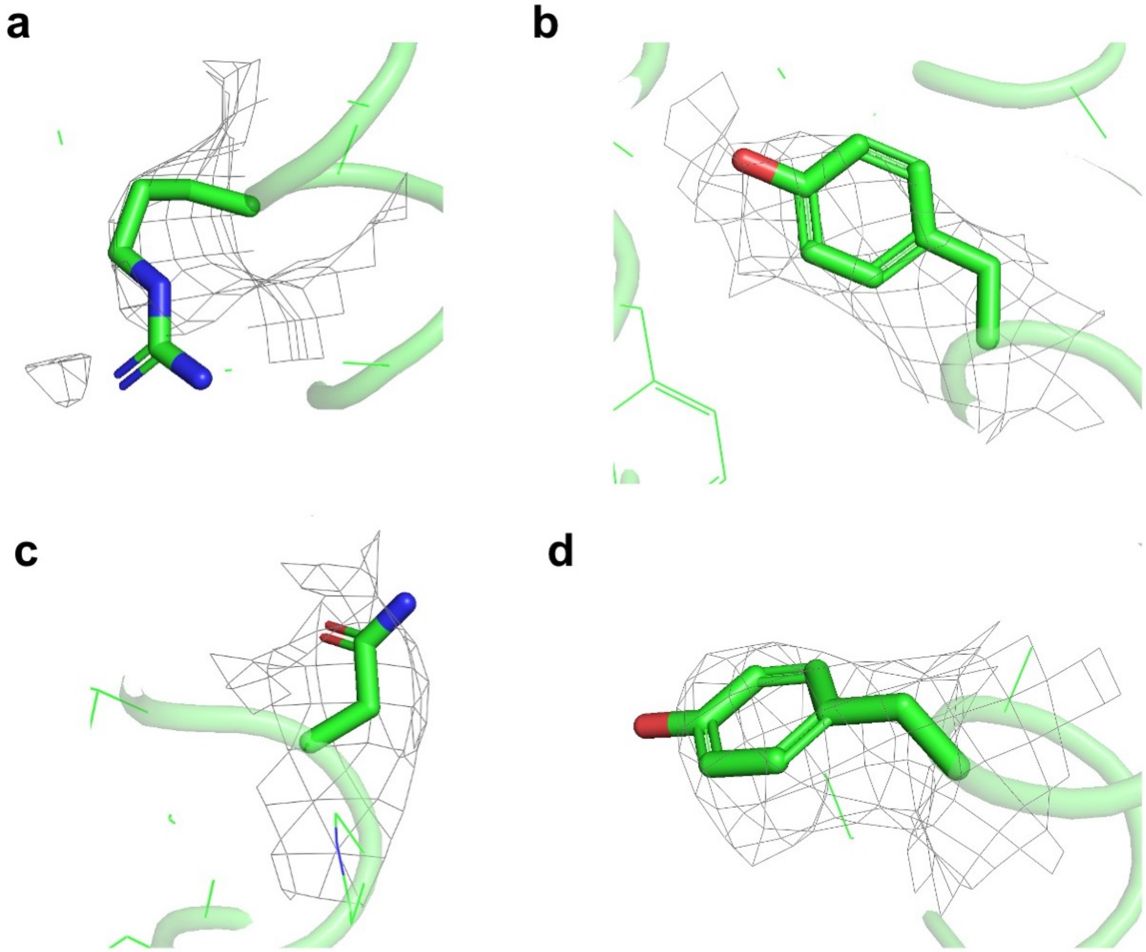

Supplementary Figure 9. Crystal contact analysis in the unite cell of Hyd_high7 X-ray structure. The receptor and T4L proteins of the original copy are colored in green and pink, respectively. The receptor and T4L proteins of the symmetry related molecules are colored in yellow and grey, respectively. The side chain of the residues involved in crystal contacts (within 4.5 Å distance) are shown in stick. **a.** view on TMHs 6 and 7. While 2 residues of TM5 make contacts with another receptor molecule, no residues of TM6 and 7 are in crystal contacts. **b.** view on TMHs 1, 2 and 3. 2 residues of intracellular 2 are in contact with a T4L molecule.

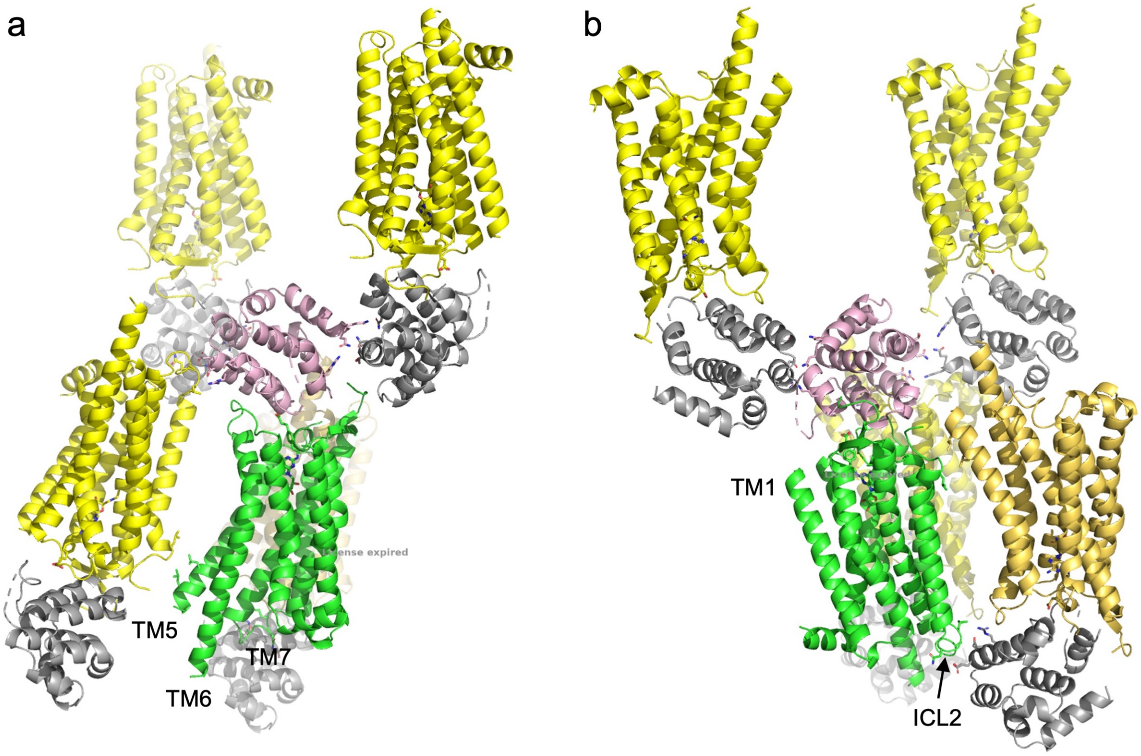

Supplementary Table 1. Amino-acid sequences of the designed receptor variants. The mutations designed using the method SPaDES in the background of the A2AR receptor are reported for each variant.

Variant	Designed mutations (Ballesteros Weinstein notation in superscript)
Hyd_low1	D52$^{2.50}$N
Hyd_low2	S91$^{3.39}$V
Hyd_low3	L95$^{3.43}$E
Hyd_high1	L48$^{2.46}$A
Hyd_high2	D52$^{2.50}$N.N284$^{7.49}$D
Hyd_high3	S91$^{3.39}$T
Hyd_high4	L95$^{3.43}$M
Hyd_high5	L95$^{3.43}$Q
Hyd_high6	I238$^{6.40}$Y
Hyd_high7	L48$^{2.48}$A.S91$^{3.39}$T.L95$^{3.43}$M.L194$^{5.55}$M.I238$^{6.40}$Y.V239$^{6.41}$L.A243$^{6.45}$L
Hyd_high8	L48$^{2.48}$A.L194$^{5.55}$M.I238$^{6.40}$Y.V239$^{6.41}$L.A243$^{6.45}$L
Hyd_high9	L48$^{2.48}$A.S91$^{3.39}$T.L95$^{3.43}$Q.L194$^{5.55}$M.I238$^{6.40}$Y.V239$^{6.41}$L.A243$^{6.45}$L
Hyd_high10	L48$^{2.48}$A.S91$^{3.39}$T.L194$^{5.55}$M.I238$^{6.40}$Y.V239$^{6.41}$L.A243$^{6.45}$L
Hyd_high11	I16$^{1.42}$N.L48$^{2.48}$A.S91$^{3.39}$T.L95$^{3.43}$M.L194$^{5.55}$M.I238$^{6.40}$Y.V239$^{6.41}$L.A243$^{6.45}$L

Supplementary Table 2. Computational metrics of the designed receptors. The table reports from left to right: change in conformational energy (inactive, active, active – inactive) from A2AR in the ligand-free (apo) state; number of water-mediated interactions between static and switchable helices; change in sodium interaction energy from A2AR in the active state; change in sodium interaction energy from A2AR in the inactive state. Reported values are taken from the lowest energy structural model in each state.

Class of designs	designs	$\Delta G_{inactive}$ from A2AR (REU)	ΔG_{active} from A2AR (REU)	$\Delta\Delta G_{apo}$ (active - inactive) from A2AR (REU)	# water-mediated static-switchable hbonds	ΔNa+ interaction energy (active) from A2AR (REU)	ΔNa+ interaction energy (inactive) from A2AR (REU)
	A2AR WT	0	0	0	20	0	0
Hyd_low	Hyd_low1	1.10	3.87	2.77	14	-0.23	0.36
	Hyd_low2	1.04	0.40	-0.64	14	0.06	0.49
	Hyd_low3	1.40	3.20	1.80	16	-0.74	-0.18
Hyd_high	Hyd_high1	1.60	4.37	2.77	22	0.04	0.11
	Hyd_high2	0.56	3.11	2.55	24	0.35	0.51
	Hyd_high3	-1.01	-0.70	0.31	26	-0.73	0.01
	Hyd_high4	2.13	0.79	-1.34	21	-0.17	0.17
	Hyd_high5	-0.16	4.10	4.22	22	-0.39	-0.18
	Hyd_high6	10.17	1.02	-9.14	25	0.16	-0.12
	Hyd_high7	9.37	4.90	-4.47	25	0.30	0.41
	Hyd_high8	15.20	3.54	-11.66	24	-0.14	-0.14
	Hyd_high9	15.28	6.91	-8.37	26	-0.30	0.33
	Hyd_high10	18.22	-0.95	-19.18	24	-0.10	0.06
	Hyd_high11	20.89	4.83	-16.06	26	-0.36	-0.13

Supplementary Table 3. Computational metrics of the top 10 models for each receptor. The table reports from left to right: change in conformational energy (inactive, active, active – inactive) from A2AR in the ligand-free (apo) state (mean ± standard error of the top 10 lowest energy structures); change in sodium interaction energy from A2AR in the active state; change in sodium interaction energy from A2AR in the inactive state (mean ± standard error of the top 10 lowest energy structures).

Class of designs	designs	Mean $\Delta G_{inactive}$ from A2AR (REU) over Top10 models ± sterr (mean)	Mean ΔG_{active} from A2AR (REU) over Top10 models ± sterr (mean)	Mean $\Delta\Delta G_{apo}$ (active - inactive) from A2AR (REU) over Top10 models ± sterr (mean)	Mean ΔNa+ interaction energy (active) from A2AR (REU) over Top10 models ± sterr (mean)	Mean ΔNa+ interaction energy (inactive) from A2AR (REU) over Top10 models ± sterr (mean)
	A2AR WT	0	0	0	0	0
Hyd_low	Hyd_low1	0.89 ± 0.25	4.17 ± 0.16	3.27 ± 0.30	-0.20 ± 0.01	0.30 ± 0.02
	Hyd_low2	-0.04 ± 0.26	0.32 ± 0.17	0.36 ± 0.31	0.13 ± 0.02	0.41 ± 0.02
	Hyd_low3	1.16 ± 0.36	3.05 ± 0.18	1.89 ± 0.41	-0.75 ± 0.01	-0.18 ± 0.02
Hyd_high	Hyd_high1	2.01 ± 0.38	4.34 ± 0.21	2.33 ± 0.43	-0.11 ± 0.04	0.09 ± 0.01
	Hyd_high2	0.80 ± 0.35	3.56 ± 0.19	2.76 ± 0.40	0.35 ± 0.01	0.54 ± 0.02
	Hyd_high3	0.27 ± 0.30	-0.49 ± 0.15	-0.76 ± 0.33	-0.82 ± 0.02	0.02 ± 0.02
	Hyd_high4	2.73 ± 0.24	0.73 ± 0.20	-2.00 ± 0.31	-0.16 ± 0.01	0.14 ± 0.02
	Hyd_high5	0.04 ± 0.26	3.33 ± 0.18	3.29 ± 0.32	-0.39 ± 0.00	-0.14 ± 0.02
	Hyd_high6	10.60 ± 0.28	1.42 ± 0.18	-9.18 ± 0.33	0.16 ± 0.00	-0.11 ± 0.01
	Hyd_high7	12.19 ± 0.45	4.26 ± 0.25	-7.94 ± 0.52	0.28 ± 0.01	0.35 ± 0.02
	Hyd_high8	14.09 ± 0.31	4.49 ± 0.30	-9.60 ± 0.43	-0.12 ± 0.01	-0.10 ± 0.02
	Hyd_high9	17.30 ± 0.41	7.22 ± 0.31	-10.08 ± 0.51	-0.29 ± 0.02	0.25 ± 0.03
	Hyd_high10	17.63 ± 0.29	-0.32 ± 0.17	-17.95 ± 0.34	0.01 ± 0.01	0.02 ± 0.01
	Hyd_high11	20.41 ± 0.32	5.38 ± 0.31	-15.04 ± 0.44	-0.48 ± 0.02	-0.34 ± 0.01

Supplementary Table 4. Summary of experimentally measured properties of designed variants. The table reports from left to right the experimentally measured change in constitutive activity (percent of fully adenosine induced A2AR activity), standard deviation of the mean constitutive activity, maximal adenosine agonist induced activity (normalized to fully adenosine induced A2AR activity), standard deviation of the mean induced activity, measured half-life of active state, standard deviation of the mean half-life of active state. N.D. not measurable

Class	Variant	Constitutive activity (%) normalized to fully induced WT activity	Stdev %CA	Adenosine-induced Activity (%) normalized to fully induced WT activity	stdev %IA	Stability (Half-life of induced activity at 37 deg C), min	stdev half life (min)
WT	A2AR	5.63	3.87	100	4.50	28.7	0.6
Hyd_low	Hyd_low1	1.30	2.72	22.72	1.52	27.2	2.2
	Hyd_low2	5.25	2.70	56.60	4.90	27.7	1.3
	Hyd_low3	10.27	7.73	16.81	0.21	NA	NA
Hyd_high	Hyd_high1	7.23	2.34	101.87	1.95	42.9	1.2
	Hyd_high2	13.90	2.38	116.93	4.10	30.8	0.7
	Hyd_high3	17.05	2.46	118.92	2.99	34.6	0.6
	Hyd_high4	12.81	2.51	95.28	4.35	38.2	0.8
	Hyd_high5	12.65	2.65	102.54	0.64	38.3	0.5
	Hyd_high6	24.02	7.84	112.14	6.57	ND	ND
	Hyd_high7	37.72	2.78	97.26	2.63	43.7	0.8
	Hyd_high8	33.67	2.63	100.66	2.93	41.3	0.7
	Hyd_high9	35.61	2.86	90.32	1.10	40.3	0.5
	Hyd_high10	37.23	2.77	96.28	4.31	42.3	0.5
	Hyd_high11	38.10	6.57	94.19	4.09	40.4	0.9

Supplementary Table 5. Adenosine ligand binding properties of selected Hyd_high designed receptors. Measured dissociation constants (mean and stdev) for [3H]-adenosine binding to purified receptors are reported.

**Variant**	**[3H]-adenosine binding Kd (nM)**	**Stdev Kd**
A2AR WT	62.6	0.6
Hyd_high1	53.1	2.8
Hyd_high4	55	2
Hyd_high7	55.8	15.7
Hyd_high9	32.5	1.8
Hyd_high10	34.7	1.6
Hyd_high11	30	1.9

Supplementary Table 6. Effects of sodium ion concentration on the activation properties of Hyd_high variants. The table reports from left to right the experimentally measured change in constitutive activity (percent of fully adenosine induced WT activity), standard deviation of the mean constitutive activity, maximal adenosine agonist induced activity (normalized to fully adenosine induced WT activity), standard deviation of the mean induced activity, measured half-life of active state, standard deviation of the mean half-life of active state, apparent melting temperature of adenosine bound receptor in high or low sodium ion conditions, standard deviation of the mean apparent melting temperature.

Na+ binding properties	Variant	Apparent Tm (deg C) (100mM NaCl)	stdev Tm (deg C) (100mM NaCl)	Apparent Tm (deg C) (100mM KCl)	stdev Tm (deg C) (100mM KCl)
High	A2AR WT	27.6	0.3	29	0.5
	Hyd_high8	39.8	0.5	43.5	0.5
Low	Hyd_high7	41.7	0.5	41.6	0.6
	Hyd_high9	41.7	0.5	41.6	0.4
	Hyd_high10	41.8	0.5	39.6	0.4

Supplementary Table 7. Data Collection and Refinement Statistics for the T4Lcp-Hyd_high7 structure.

	Hyd_high7
**Data collection**	
Space group	1
Cell dimensions	$P2_12_12_1$
a, *b*, *c* (Å)	60.18, 73.67, 149.85
α, β, γ (°)	90.00, 90.00, 90.00
Resolution (Å)	66.00-3.9 (4.03-3.9) *
R_{sym} or R_{merge}	0.313(1.634)
$I / \sigma I$	7.3 (1.9)
Completeness (%)	99.3 (99.8)
Redundancy	6.3 (6.7)
**Refinement**	
Resolution (Å)	24.97-3.9
No. reflections	6371
R_{work} / R_{free}	0.275 / 0.293
No. atoms	
Protein	3020
Ligand/ion	36
B-factors	
Protein	124
Ligand/ion	113
R.m.s. deviations	
Bond lengths (Å)	0.012
Bond angles (°)	1.37

*Number of xtals for each structure should be noted in footnote. *Values in parentheses are for highest-resolution shell.

Supplementary Table 8. Comparison between Phenix.SPaDES_refine and Phenix-Rosetta.refine structure refinement softwares. Model quality and statistics of the Hyd_high7 structures refined using Phenix.SPaDES_refine (above) or Phenix.SPaDES_refine (bottom).

<u>Phenix-Spades:</u>

Ramachandran outliers	=	0.00 %
favored	=	93.33 %
Rotamer outliers	=	0.00 %
C-beta deviations	=	0
Clashscore	=	2.12
RMS(bonds)	=	0.0027
RMS(angles)	=	0.66
MolProbity score	=	1.42
Resolution	=	3.90
R-work	=	0.2864
R-free	=	0.3240

<u>Phenix-Rosetta:</u>

Ramachandran outliers	=	0.53 %
favored	=	94.93 %
Rotamer outliers	=	0.31 %
C-beta deviations	=	0
Clashscore	=	2.45
RMS(bonds)	=	0.0031
RMS(angles)	=	0.72
MolProbity score	=	1.38
Resolution	=	3.90
R-work	=	0.3021
R-free	=	0.3453

Supplementary Table 9. Conservation of solvated sites between High7 and native A2A and mOR receptors. a. Conservation of solvated sites (i.e. contacting a water molecule) between mOR and High7 active state structures (see **Fig.5**). bb and sc denote backbone and side-chain solvated sites, respectively. NE: not existent. **b.** Conservation of solvated residues among natural A2AR and mOR sequences.

a.

mOR	High7	Common positions	Common sites
N1.50 (sc)	N1.50 (sc)	1	1
I2.43 (bb)	NE	0	0
D2.50 (sc)	D2.50 (sc)	1	1
S3.39 (sc)	NE	0	0
L3.43 (bb)	NE	0	0
Y5.58 (sc)	NE	0	0
V6.40 (bb)	Y6.40 (bb)	1	1
NE	Y6.40 (sc)	0	0
W6.48 (sc)	W6.48 (sc)	1	1
N7.45 (sc)	N7.45 (sc)	1	1
NE	N7.49 (bb)	0	0
N7.49 (sc)	N7.49 (sc)	1	1
NE	F7.51 (bb)	0	0
NE	Y7.53 (bb)	1	0
Y7.53 (sc)	NE	0	0
Conservation		7/12 = 58%	6/15 = 40%

b.

mOR	A2AR	% mOR residue conservation	% A2AR residue conservation
N1.50 (sc)	N1.50	100	100
I2.43 (bb)	V2.43	74	12
D2.50 (sc)	D2.50	100	100
S3.39 (sc)	S3.39	100	100
L3.43 (bb)	L3.43	100	100
Y5.58 (sc)	Y5.58	100	100
V6.40 (bb)	I6.40	58	42
W6.48 (sc)	W6.48	98	98
N7.45 (sc)	N7.45	98	98
N7.49 (sc)	N7.49	100	100
Y7.53 (sc)	Y7.53	100	100

Supplementary Table 10. Conservation of designed residues in class A GPCRs. Conservation of native human A2AR residue and occurrence of designed mutations within Adenosine+opioid receptor orthologs and within all class A GPCRs with an e-value < $1e^{-2}$ to human A2AR.

Native A2AR residue		Designed mutation	Adenosine + opioid receptors		Class A GPCRs < $1E^{-2}$ E-value to human A2AR	
Position (BW)	Position (uniprot)		% Residue conservation	% Mutation occurrence	% Residue conservation	% Mutation occurrence
I1.42	I16	N	56	0	28.8	0
L2.46	L48	A	100	0	93.8	0
D2.50	D52	N	100	0	92.4	4.6
S3.39	S91	V	100	0	72.4	0
S3.39	S91	T	100	0	72.4	5.0
L3.43	L95	Q	100	0	77.0	0
L3.43	L95	E	100	0	77.0	0
L3.43	L95	M	100	0	77.0	5.7
L5.55	L194	M	26	2	18.4	0.9
I6.40	I238	Y	42	0	30.1	0.5
V6.41	V239	L	68	32	40.6	22.8
A6.45	A243	L	58	0	13.9	20.7
N7.49	N284	D	100	0	79.5	16.1
Average conservation			80.8	2.6	59.5	5.9

Supplementary Table 11. Occurrence of combined mutations from the designed variants Hyd_high7 to 11 within all class A GPCRs. Motifs not reported were not observed. Full designed Hyd_high7 to 11 sequences could not be found in class A GPCRs.

Designed Motif	% occurrence in class A GPCRs
N2.50, D7.49	0.95
T3.39, M3.43	0.06
T3.39, L6.41	0.19
T3.39, L6.45	0.63
M3.43, L6.45	0.63
M5.55, L6.45	0.13
M5.55, L6.41, L6.45	0.06
L6.41, L6.45	1.77
Y6.40, L6.45	0.25
Hyd_high7	0
Hyd_high8	0
Hyd_high9	0
Hyd_high10	0
Hyd_high11	0

Supplementary Discussion

Below, we provide additional analysis of the designed variants concerning 1) the mechanistic insights into water-mediated allostery and 2) solvent-mediated interactions

<u>*1. Mechanistic insights into water-mediated allostery for designed variants at hydrophobic residues*</u>

We investigated the role of hydrophobic residues lining the hydrated cavity on the solvent-mediated polar network and signal transduction by targeting positions 2.46 and 3.43 located one helical turn below Asp 2.50 and Ser 3.39, respectively.

Hyd_low3 introduces a negatively charged Glu at position 3.43, one helical turn below Ser 3.39, that perturbs the solvent-mediated network by removing the hydrophobic gateway between native Leu 3.43 and Leu 2.46 that separates the solvated cavity from the active state intracellular side. The designed mutation significantly decreased the overall hydration between static and switchable helices (**Supplementary Table 2**) and disturbed the water-mediated allosteric network topology near the toggle switch Trp 6.48. In particular, Glu 3.43 forms 2 non-native water-mediated interactions with Trp 6.48 and Asn 7.45, the latter abrogating the highly conserved active state contact between Ser 3.36 and Asn 7.45 (**Extended Data Figure 4**). In line with the loss of hydration and breaking of key activating contacts, ligand-induced activation of the designed receptor was only 17% versus A2AR (**Supplementary Table 4**).

To better understand the impact of the side-chain chemistry at site 3.43, we tried both Met 3.43 as a hydrophobic control and Gln 3.43 as an uncharged polar residue. Both residues increased hydration between static and switchable helices and were therefore designated as Hyd_high4 and Hyd_high5 (**Supplementary Table 2**). Met occupies more space in the

cavity than Leu, preventing a water-mediated contact between Ser 3.36 and Asn 7.45 but enabling a new solvated interaction with Leu 6.46, while the polar network remained unperturbed in the vicinity of Asp 2.50 (**Extended Data Figure 5**). Overall the altered topology provided a higher conformational stability of the active state, a finding validated through enhanced constitutive activity and lifetime of the active state, but unaffected ligand-induced activity compared with A2AR. (**Supplementary Table 4**). Unlike Met, the polar side-chain of Gln at 3.43 in Hyd_high5 perturbs the polar network proximal to the toggle switch. It directly interacts with Trp 6.48 through a new strong water-mediated interaction, thereby breaking a weak polar contact between Trp 6.48 and Asn 7.45 and triggering the formation of a water-mediated polar bond between Asn 7.45 and Leu 6.46 (**Extended Data Figure 6**). Asn 7.45 ligand induced activity was unaffected, suggesting that receptor activation can accommodate the polar network rewiring at the toggle switch. Constitutive activity and lifetime of the active state were higher than A2AR, agreeing with our predictions (**Figure 3d**, **Supplementary Table 4**).

We next tested the impact of breaking the hydrophobic gateway at Leu 2.46 via the Hyd_high1 design where Leu is substituted for Ala. The new microcavity one helical turn below Asp 2.50 creates a new intra-TMH2 water-mediated contact between Asp 2.50 and Ala 2.46. This additional microcavity subtly reorganized water molecule conformations within the network while the overall topology remained similar to A2AR (**Extended Data Figure 7**). Consistent with these small allosteric network perturbations, adenosine-induced Hyd_high1 activity was largely unaffected (**Supplementary Table 4**). Constitutive activity however was higher, in line with the measured enhanced lifetime of the active state.

2. Impact of the designed mutations onto ion-mediated conformational stability and receptor activity.

Structural analysis revealed a number a design-specific effects on solvent-mediated network topologies and Na+ binding propensities. From our Na+ binding simulations, we extracted ion occupancies in distinct states of the receptors and validated them using thermal shift assays in presence of either Na+ or K+. Because K+ is substantially larger, it cannot be well accommodated in the Na+ binding site of GPCRs. In A2AR, Na+ preferentially binds to the inactive state, implying that the absence of this ion should enhance the conformational stability of the active state over the inactive. Consistent with these expectations, the apparent melting temperature of A2AR active state rose by 4°C when Na+ was replaced by K+. We found that while Hyd_high8 active state stability was dependent on Na+ similarly to A2AR, the other designs lost sensitivity to the ion (**Supplementary Table 6**). To gain deeper structural understanding of these effects, we calculated the impacts of the designs from our Rosetta-based ion binding simulations on 1) the overall Na+ binding propensity and 2) the preferential Na+ occupancy in the inactive versus active state (**Methods**, **Supplementary Fig. 4**). While the Boltzmann distributions derived from Rosetta energies not an accurate representation of any true thermodynamics, they should provide the insight necessary to qualitatively discuss the relative ion occupancies in the context of the experimentally measured responses.

Compared to A2AR, Hyd_high8 was characterized as having a substantially higher overall Na+ binding propensity and a mild decrease in preferential inactive state occupancy. Hence, Na+ binds strongly to the receptor and favors the inactive state similarly to A2AR, in agreement with the experiments. Hyd_high7 possessed a large decrease in overall Na+ binding and loss in preferential inactive state occupancy, and we subsequently found a loss in measured sensitivity to Na+ and higher basal activity. Calculations of Hyd_high9

were also consistent with the experiments but revealed that the lack of Na+ sensitivity stemmed primarily from a very large decrease in preferential inactive state occupancy. While the calculated effects were less pronounced for Hyd_high10, the weakened Na+ binding propensity was also consistent with the experiment. The overall qualitative agreement between predictions and experiments suggests that our approach captures the important determinants of conformationally selective receptor-ion interactions.

Supplementary Data

Designed receptor sequences

WT (from uniprot P29274)
MPIMGSSVYITVELAIAVLAILGNVLVCWAVWLNSNLQNVTNYFVVSLAAADIAVGVLAI
PFAITISTGFCAACHGCLFIACFVLVLTQSSIFSLLAIAIDRYIAIRIPLRYNGLVTGTR
AKGIIAICWVLSFAIGLTPMLGWNNCGQPKEGKNHSQGCGEGQVACLFEDVVPMNYMVYF
NFFACVLVPLLLMLGVYLRIFLAARRQLKQMESQPLPGERARSTLQKEVHAAKSLAIIVG
LFALCWLPLHIINCFTFFCPDCSHAPLWLMYLAIVLSHTNSVVNPFIYAYRIREFRQTFR
KIIRSHVLRQQEPFKAAGTSARVLAAHGSDGEQVSLRLNGHPPGVWANGSAPHPERRPNG
YALGLVSGGSAQESQGNTGLPDVELLSHELKGVCPEPPGLDDPLAQDGAGVS

Key: ICL, ECL, **Mutation**

Low1 (D2.50N / D52N)
MPIMGSSVYITVELAIAVLAILGNVLVCWAVWLNSNLQNVTNYFVVSLAAA**N**IAVGVLAI
PFAITISTGFCAACHGCLFIACFVLVLTQSSIFSLLAIAIDRYIAIRIPLRYNGLVTGTR
AKGIIAICWVLSFAIGLTPMLGWNNCGQPKEGKNHSQGCGEGQVACLFEDVVPMNYMVYF
NFFACVLVPLLLMLGVYLRIFLAARRQLKQMESQPLPGERARSTLQKEVHAAKSLAIIVG
LFALCWLPLHIINCFTFFCPDCSHAPLWLMYLAIVLSHTNSVVNPFIYAYRIREFRQTFR
KIIRSHVLRQQEPFKAAGTSARVLAAHGSDGEQVSLRLNGHPPGVWANGSAPHPERRPNG
YALGLVSGGSAQESQGNTGLPDVELLSHELKGVCPEPPGLDDPLAQDGAGVS

Low2 (S3.39V / S91V)
MPIMGSSVYITVELAIAVLAILGNVLVCWAVWLNSNLQNVTNYFVVSLAAADIAVGVLAI
PFAITISTGFCAACHGCLFIACFVLVLTQS**V**IFSLLAIAIDRYIAIRIPLRYNGLVTGTR
AKGIIAICWVLSFAIGLTPMLGWNNCGQPKEGKNHSQGCGEGQVACLFEDVVPMNYMVYF
NFFACVLVPLLLMLGVYLRIFLAARRQLKQMESQPLPGERARSTLQKEVHAAKSLAIIVG
LFALCWLPLHIINCFTFFCPDCSHAPLWLMYLAIVLSHTNSVVNPFIYAYRIREFRQTFR
KIIRSHVLRQQEPFKAAGTSARVLAAHGSDGEQVSLRLNGHPPGVWANGSAPHPERRPNG
YALGLVSGGSAQESQGNTGLPDVELLSHELKGVCPEPPGLDDPLAQDGAGVS

Low3 (L3.43E / L95E)
MPIMGSSVYITVELAIAVLAILGNVLVCWAVWLNSNLQNVTNYFVVSLAAADIAVGVLAI
PFAITISTGFCAACHGCLFIACFVLVLTQSSIFS**E**LAIAIDRYIAIRIPLRYNGLVTGTR
AKGIIAICWVLSFAIGLTPMLGWNNCGQPKEGKNHSQGCGEGQVACLFEDVVPMNYMVYF
NFFACVLVPLLLMLGVYLRIFLAARRQLKQMESQPLPGERARSTLQKEVHAAKSLAIIVG
LFALCWLPLHIINCFTFFCPDCSHAPLWLMYLAIVLSHTNSVVNPFIYAYRIREFRQTFR
KIIRSHVLRQQEPFKAAGTSARVLAAHGSDGEQVSLRLNGHPPGVWANGSAPHPERRPNG
YALGLVSGGSAQESQGNTGLPDVELLSHELKGVCPEPPGLDDPLAQDGAGVS

High1 (L2.46A / L48A)
MPIMGSSVYITVELAIAVLAILGNVLVCWAVWLNSNLQNVTNYFVVS**A**AAADIAVGVLAI
PFAITISTGFCAACHGCLFIACFVLVLTQSSIFSLLAIAIDRYIAIRIPLRYNGLVTGTR
AKGIIAICWVLSFAIGLTPMLGWNNCGQPKEGKNHSQGCGEGQVACLFEDVVPMNYMVYF
NFFACVLVPLLLMLGVYLRIFLAARRQLKQMESQPLPGERARSTLQKEVHAAKSLAIIVG
LFALCWLPLHIINCFTFFCPDCSHAPLWLMYLAIVLSHTNSVVNPFIYAYRIREFRQTFR
KIIRSHVLRQQEPFKAAGTSARVLAAHGSDGEQVSLRLNGHPPGVWANGSAPHPERRPNG

YALGLVSGGSAQESQGNTGLPDVELLSHELKGVCPEPPGLDDPLAQDGAGVS

High2 (D2.50N, N7.49D / D52N, N284D)
MPIMGSSVYITVELAIAVLAILGNVLVCWAVWLNSNLQNVTNYFVVSLAAA**N**IAVGVLAI
PFAITISTGFCAACHGCLFIACFVLVLTQSSIFSLLAIAIDRYIAIRIPLRYNGLVTGTR
AKGIIAICWVLSFAIGLTPMLGWNNCGQPKEGKNHSQGCGEGQVACLFEDVVPMNYMVYF
NFFACVLVPLLLMLGVYLRIFLAARRQLKQMESQPLPGERARSTLQKEVHAAKSLAIIVG
LFALCWLPLHIINCFTFFCPDCSHAPLWLMYLAIVLSHTNSVV**D**PFIYAYRIREFRQTFR
KIIRSHVLRQQEPFKAAGTSARVLAAHGSDGEQVSLRLNGHPPGVWANGSAPHPERRPNG
YALGLVSGGSAQESQGNTGLPDVELLSHELKGVCPEPPGLDDPLAQDGAGVS

High3 (S3.39T / S91T)
MPIMGSSVYITVELAIAVLAILGNVLVCWAVWLNSNLQNVTNYFVVSLAAADIAVGVLAI
PFAITISTGFCAACHGCLFIACFVLVLTQS**T**IFSLLAIAIDRYIAIRIPLRYNGLVTGTR
AKGIIAICWVLSFAIGLTPMLGWNNCGQPKEGKNHSQGCGEGQVACLFEDVVPMNYMVYF
NFFACVLVPLLLMLGVYLRIFLAARRQLKQMESQPLPGERARSTLQKEVHAAKSLAIIVG
LFALCWLPLHIINCFTFFCPDCSHAPLWLMYLAIVLSHTNSVVNPFIYAYRIREFRQTFR
KIIRSHVLRQQEPFKAAGTSARVLAAHGSDGEQVSLRLNGHPPGVWANGSAPHPERRPNG
YALGLVSGGSAQESQGNTGLPDVELLSHELKGVCPEPPGLDDPLAQDGAGVS

High4 (L3.43M / L95M)
MPIMGSSVYITVELAIAVLAILGNVLVCWAVWLNSNLQNVTNYFVVSLAAADIAVGVLAI
PFAITISTGFCAACHGCLFIACFVLVLTQSSIFS**M**LAIAIDRYIAIRIPLRYNGLVTGTR
AKGIIAICWVLSFAIGLTPMLGWNNCGQPKEGKNHSQGCGEGQVACLFEDVVPMNYMVYF
NFFACVLVPLLLMLGVYLRIFLAARRQLKQMESQPLPGERARSTLQKEVHAAKSLAIIVG
LFALCWLPLHIINCFTFFCPDCSHAPLWLMYLAIVLSHTNSVVNPFIYAYRIREFRQTFR
KIIRSHVLRQQEPFKAAGTSARVLAAHGSDGEQVSLRLNGHPPGVWANGSAPHPERRPNG
YALGLVSGGSAQESQGNTGLPDVELLSHELKGVCPEPPGLDDPLAQDGAGVS

High5 (L3.43Q / L95Q)
MPIMGSSVYITVELAIAVLAILGNVLVCWAVWLNSNLQNVTNYFVVSLAAADIAVGVLAI
PFAITISTGFCAACHGCLFIACFVLVLTQSSIFS**Q**LAIAIDRYIAIRIPLRYNGLVTGTR
AKGIIAICWVLSFAIGLTPMLGWNNCGQPKEGKNHSQGCGEGQVACLFEDVVPMNYMVYF
NFFACVLVPLLLMLGVYLRIFLAARRQLKQMESQPLPGERARSTLQKEVHAAKSLAIIVG
LFALCWLPLHIINCFTFFCPDCSHAPLWLMYLAIVLSHTNSVVNPFIYAYRIREFRQTFR
KIIRSHVLRQQEPFKAAGTSARVLAAHGSDGEQVSLRLNGHPPGVWANGSAPHPERRPNG
YALGLVSGGSAQESQGNTGLPDVELLSHELKGVCPEPPGLDDPLAQDGAGVS

High6 (I6.40Y / I238Y)
MPIMGSSVYITVELAIAVLAILGNVLVCWAVWLNSNLQNVTNYFVVSLAAADIAVGVLAI
PFAITISTGFCAACHGCLFIACFVLVLTQSSIFSLLAIAIDRYIAIRIPLRYNGLVTGTR
AKGIIAICWVLSFAIGLTPMLGWNNCGQPKEGKNHSQGCGEGQVACLFEDVVPMNYMVYF
NFFACVLVPLLLMLGVYLRIFLAARRQLKQMESQPLPGERARSTLQKEVHAAKSLAI**Y**VG
LFALCWLPLHIINCFTFFCPDCSHAPLWLMYLAIVLSHTNSVVNPFIYAYRIREFRQTFR
KIIRSHVLRQQEPFKAAGTSARVLAAHGSDGEQVSLRLNGHPPGVWANGSAPHPERRPNG
YALGLVSGGSAQESQGNTGLPDVELLSHELKGVCPEPPGLDDPLAQDGAGVS

High7 (L2.46A, S3.39T, L3.43M, L5.55M, I6.40Y, V6.41L, A6.45L / L48A, S91T, L95M, L194M, I238Y, V239L, A243L)
MPIMGSSVYITVELAIAVLAILGNVLVCWAVWLNSNLQNVTNYFVVS**A**AAADIAVGVLAI

PFAITISTGFCAACHGCLFIACFVLVLTQS**T**IFS**M**LAIAIDRYIAIRIPLRYNGLVTGTR
AKGIIAICWVLSFAIGLTPMLGWNNCGQPKEGKNHSQGCGEGQVACLFEDVVPMNYMVYF
NFFACVLVPLLLM**M**GVYLRIFLAARRQLKQMESQPLPGERARSTLQKEVHAAKSLAI**YL**G
LF**L**LCWLPLHIINCFTFFCPDCSHAPLWLMYLAIVLSHTNSVVNPFIYAYRIREFRQTFR
KIIRSHVLRQQEPFKAAGTSARVLAAHGSDGEQVSLRLNGHPPGVWANGSAPHPERRPNG
YALGLVSGGSAQESQGNTGLPDVELLSHELKGVCPEPPGLDDPLAQDGAGVS

High8 (L2.46A, L5.55M, I6.40Y, V6.41L, A6.45L / L48A, L194M, I238Y, V239L, A243L)
MPIMGSSVYITVELAIAVLAILGNVLVCWAVWLNSNLQNVTNYFVVS**A**AAADIAVGVLAI
PFAITISTGFCAACHGCLFIACFVLVLTQSSIFSLLAIAIDRYIAIRIPLRYNGLVTGTR
AKGIIAICWVLSFAIGLTPMLGWNNCGQPKEGKNHSQGCGEGQVACLFEDVVPMNYMVYF
NFFACVLVPLLLM**M**GVYLRIFLAARRQLKQMESQPLPGERARSTLQKEVHAAKSLAI**YL**G
LF**L**LCWLPLHIINCFTFFCPDCSHAPLWLMYLAIVLSHTNSVVNPFIYAYRIREFRQTFR
KIIRSHVLRQQEPFKAAGTSARVLAAHGSDGEQVSLRLNGHPPGVWANGSAPHPERRPNG
YALGLVSGGSAQESQGNTGLPDVELLSHELKGVCPEPPGLDDPLAQDGAGVS

High9 (L2.46A, S3.39T, L3.43Q, L5.55M, I6.40Y, V6.41L, A6.45L / L48A, S91T, L95q, L194M, I238Y, V239L, A243L)
MPIMGSSVYITVELAIAVLAILGNVLVCWAVWLNSNLQNVTNYFVVS**A**AAADIAVGVLAI
PFAITISTGFCAACHGCLFIACFVLVLTQS**T**IFS**Q**LAIAIDRYIAIRIPLRYNGLVTGTR
AKGIIAICWVLSFAIGLTPMLGWNNCGQPKEGKNHSQGCGEGQVACLFEDVVPMNYMVYF
NFFACVLVPLLLM**M**GVYLRIFLAARRQLKQMESQPLPGERARSTLQKEVHAAKSLAI**YL**G
LF**L**LCWLPLHIINCFTFFCPDCSHAPLWLMYLAIVLSHTNSVVNPFIYAYRIREFRQTFR
KIIRSHVLRQQEPFKAAGTSARVLAAHGSDGEQVSLRLNGHPPGVWANGSAPHPERRPNG
YALGLVSGGSAQESQGNTGLPDVELLSHELKGVCPEPPGLDDPLAQDGAGVS

High10 (L2.46A, S3.39T, L5.55M, I6.40Y, V6.41L, A6.45L / L48A, S91T, L194M, I238Y, V239L, A243L)
MPIMGSSVYITVELAIAVLAILGNVLVCWAVWLNSNLQNVTNYFVVS**A**AAADIAVGVLAI
PFAITISTGFCAACHGCLFIACFVLVLTQS**T**IFSLLAIAIDRYIAIRIPLRYNGLVTGTR
AKGIIAICWVLSFAIGLTPMLGWNNCGQPKEGKNHSQGCGEGQVACLFEDVVPMNYMVYF
NFFACVLVPLLLM**M**GVYLRIFLAARRQLKQMESQPLPGERARSTLQKEVHAAKSLAI**YL**G
LF**L**LCWLPLHIINCFTFFCPDCSHAPLWLMYLAIVLSHTNSVVNPFIYAYRIREFRQTFR
KIIRSHVLRQQEPFKAAGTSARVLAAHGSDGEQVSLRLNGHPPGVWANGSAPHPERRPNG
YALGLVSGGSAQESQGNTGLPDVELLSHELKGVCPEPPGLDDPLAQDGAGVS

High11 (I1.42N, L2.46A, S3.39T, L3.43M, L5.55M, I6.40Y, V6.41L, A6.45L / I16N, L48A, S91T, L95M, L194M, I238Y, V239L, A243L)
MPIMGSSVYITVELA**N**AVLAILGNVLVCWAVWLNSNLQNVTNYFVVS**A**AAADIAVGVLAI
PFAITISTGFCAACHGCLFIACFVLVLTQS**T**IFS**M**LAIAIDRYIAIRIPLRYNGLVTGTR
AKGIIAICWVLSFAIGLTPMLGWNNCGQPKEGKNHSQGCGEGQVACLFEDVVPMNYMVYF
NFFACVLVPLLLM**M**GVYLRIFLAARRQLKQMESQPLPGERARSTLQKEVHAAKSLAI**YL**G
LF**L**LCWLPLHIINCFTFFCPDCSHAPLWLMYLAIVLSHTNSVVNPFIYAYRIREFRQTFR
KIIRSHVLRQQEPFKAAGTSARVLAAHGSDGEQVSLRLNGHPPGVWANGSAPHPERRPNG
YALGLVSGGSAQESQGNTGLPDVELLSHELKGVCPEPPGLDDPLAQDGAGVS

Supplementary Methods

An example script for running SPaDES hydrate is given below. More information, including hyfile, nofasol etc. files, can be found at https://github.com/barth-lab/SPaDES_DESIGNS.

```
#!/bin/bash

# Hydrate location
hydrate="/path/to/rosetta/main/source/bin/hydrate.linuxgccrelease"
# Include libraries (your specific compiler may mean this path doesn't exist, change as needed)
export
LD_LIBRARY_PATH=/path/to/rosetta/main/source/build/external/release/linux/5.13/64/x86/gcc/9/defaul
t/
# Rosetta database location
DATABASE="/path/to/rosetta/main/database/"

rand=$$ # seed for rosetta
currentpwd=$(pwd)

# state = input pdb structure (active or inactive)
state=${1}
# wildtype (wt) or design (des) ?
ptype=${2}

# Make a folder to place everything
RUN=${3}
touch RUN${RUN}
mkdir RUN${RUN}
cd RUN${RUN}

# assign nofasol value (read file with commas)
nofasol="$(cat ${currentpwd}/${state}_${ptype}.nofasol)"

# Run hydrate!
$hydrate @${currentpwd}/inputs/general_flags.flags \
        -hydrate:hyfile ${currentpwd}/${state}_${ptype}.hyfile \
        -packing:resfile ${currentpwd}/${state}_${ptype}_high10.resfile \
        -in:file:spanfile ${currentpwd}/STRUCTURE.span \
        -hydrate:ignore_fa_sol_at_positions ${nofasol} \
        -database ${DATABASE} \
        -in:file:s ${currentpwd}/${state}_wt.pdb  \
        -in:file:extra_res_cen ${currentpwd}/${state}_ligand.cen.params \
        -in:file:extra_res_fa ${currentpwd}/${state}_ligand.fa.params \
        -seed_offset ${rand} \
        -out:prefix ./${state}_${ptype}/  > ${state}_${ptype}.log
```

The general flags file is as follows:

```
# Energy
-restore_pre_talaris_2013_behavior true

# Scoring
-score
 -water_hybrid_sf true
 -weights opte_mb_elec.wts

# Hydrate
-hydrate
 -water_rotamers_cap 500
 -hbond_threshold -0.5

# File management
-inout:skip_connect_info true
-inout:write_all_connect_info false
-out:nstruct 200
-overwrite

# Packing
-ignore_zero_occupancy false
-use_input_sc
-ex1
-ex2
-extrachi_cutoff 1
-in:auto_setup_metals

# Membrane
-membrane:Membed_init
-membrane::Mhbond_depth

# Constraints
-cst_weight 1.0
-cst_fa_weight 1.0
```
